# Supplementary figures and images for: Effects of drought on the abundance and distribution of non-breeding shorebirds in central California, USA
Source: PLoS One. 2020 Oct 21;15(10):e0240931. doi: 10.1371/journal.pone.0240931 (PMC7577470; doi:10.1371/journal.pone.0240931)

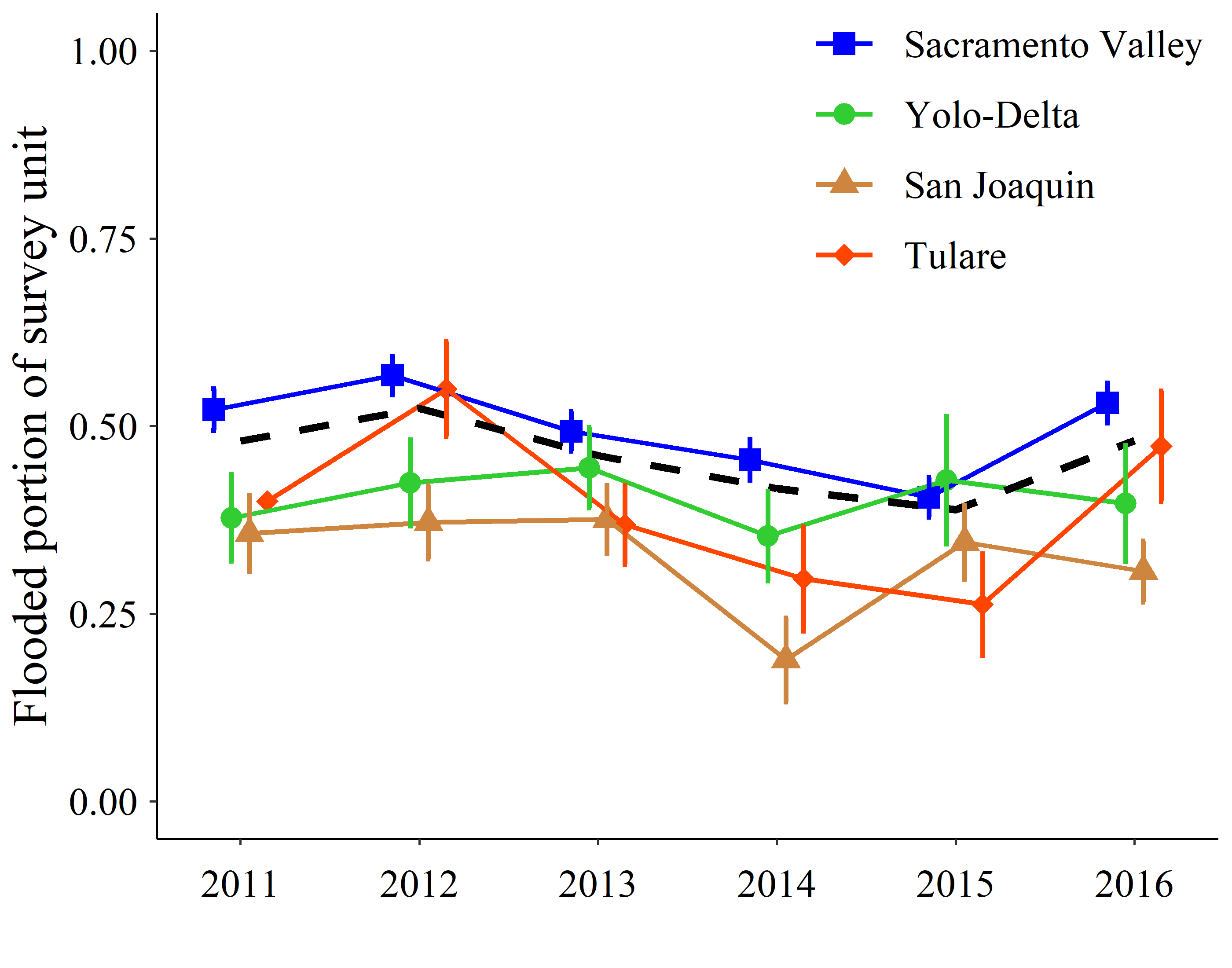

Supplement: S1 Fig — Annual means with error bars representing 95% confidence intervals from data collected during shorebird surveys from 2011 to 2016 in the Central Valley, California, USA. Survey units are categorized by the dominant land cover type within unit boundaries. The dashed line represents the annual mean estimate across all cover types. See Methods for details on the types of land cover that composed the “other suitable” and “miscellaneous” land cover categories. (TIF) [file pone.0240931.s001.tif]

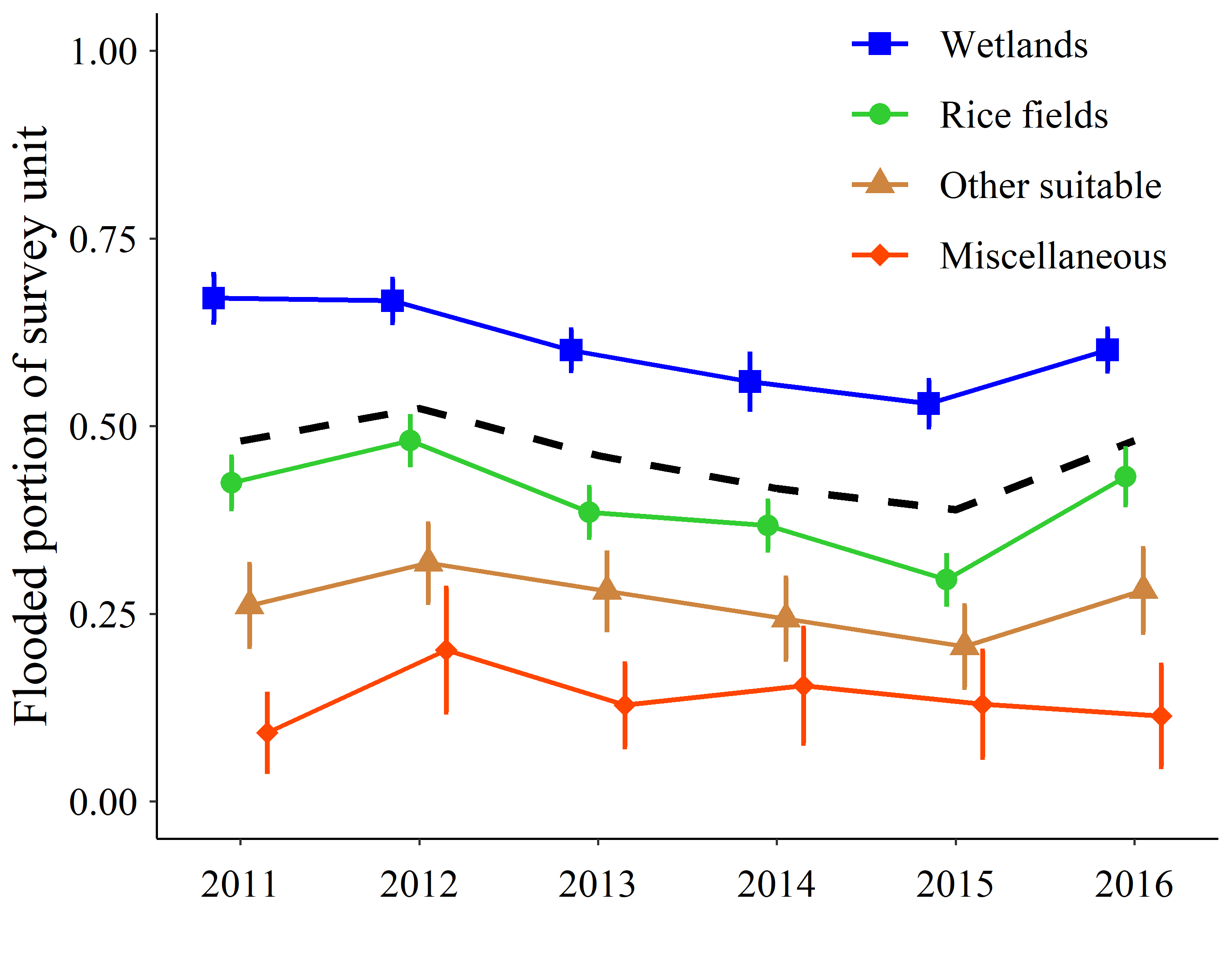

Supplement: S2 Fig — Annual means with error bars representing 95% confidence intervals from data collected during shorebird surveys from 2011 to 2016 in the Central Valley, California, USA. Survey units are categorized by geographic location within sub-regions. The dashed line represents the annual mean estimate across all cover types. (TIF) [file pone.0240931.s002.tif]
