# Supplementary material for: Effects of drought on the abundance and distribution of non-breeding shorebirds in central California, USA
Source: PLoS One. 2020 Oct 21;15(10):e0240931. doi: 10.1371/journal.pone.0240931 (PMC7577470; doi:10.1371/journal.pone.0240931)
Supplement: S1 Table — Estimated density of total shorebirds (per km2) among four sub-regions of the Central Valley and two coastal regions from 2011 to 2016 across central California, USA. We did not estimate the mean density of total shorebirds within coastal regions. Summary of our annual survey effort is located under each density estimate and displayed as the number of units and total area surveyed (km2). 1 No survey at Los Banos Wildlife Area in 2014. 2 No survey at Kern or Pixley National Wildlife Refuge in 2011 and 2014. 3 No survey of Suisun Marsh in 2011. 4 No surveys of Drakes Estero or nearby waters in 2011 and 2012. (DOCX) [file pone.0240931.s003.docx]

**S1 Table. Annual density of total shorebirds (per km^2^) and survey effort across central California, USA.**

Estimated density of total shorebirds (per km^2^) among four sub-regions of the Central Valley and two coastal regions from 2011 to 2016 across central California, USA. We did not estimate the mean density of total shorebirds within coastal regions. Summary of our annual survey effort is located under each density estimate and displayed as the number of units and total area surveyed (km^2^).

^1^ No survey at Los Banos Wildlife Area in 2014.

^2^ No survey at Kern or Pixley National Wildlife Refuge in 2011 and 2014.

^3^ No survey of Suisun Marsh in 2011.

^4^ No surveys of Drakes Estero or nearby waters in 2011 and 2012.
