# Supplementary material for: Effects of drought on the abundance and distribution of non-breeding shorebirds in central California, USA
Source: PLoS One. 2020 Oct 21;15(10):e0240931. doi: 10.1371/journal.pone.0240931 (PMC7577470; doi:10.1371/journal.pone.0240931)
Supplement: S3 Table — Estimated density of total shorebirds (per km2) among four land cover types from 2011 to 2016 in the Central Valley, California, USA. Summary of our annual survey effort is located under each density estimate and displayed as the number of units and total area surveyed (km2). See Methods for details on the types of land cover that composed the “other suitable” and “miscellaneous” land cover categories. (DOCX) [file pone.0240931.s005.docx]

**S3 Table. Annual density of total shorebirds (per km^2^) and survey effort in the Central Valley.**

Estimated density of total shorebirds (per km^2^) among four land cover types from 2011 to 2016 in the Central Valley, California, USA. Summary of our annual survey effort is located under each density estimate and displayed as the number of units and total area surveyed (km^2^). We defined “other suitable habitats” as those with potential for use by shorebirds when flooded, including grasslands/pasture, corn fields, other irrigated row or field crops, freshwater lakes/ponds, and wastewater treatment ponds. We defined “miscellaneous” land cover types as having low potential for use by shorebirds, and included developed, forested, riverine, orchard, and vineyard.
