# Supplementary material for: Effects of drought on the abundance and distribution of non-breeding shorebirds in central California, USA
Source: PLoS One. 2020 Oct 21;15(10):e0240931. doi: 10.1371/journal.pone.0240931 (PMC7577470; doi:10.1371/journal.pone.0240931)
Supplement: S6 Table — Annual density estimates from 2011 to 2016 among three regions in central California, USA. Mean annual counts are the average annual survey totals for each species without accounting for the total area surveyed. (DOCX) [file pone.0240931.s008.docx]

**S6 Table. Annual density (per km^2^) of focal shorebirds and mean annual count in central California, USA.**

Annual density estimates from 2011 to 2016 among three regions in central California, USA. Mean annual counts are the average annual survey totals for each species without accounting for the total area surveyed.
