# Supplementary material for: Effects of drought on the abundance and distribution of non-breeding shorebirds in central California, USA
Source: PLoS One. 2020 Oct 21;15(10):e0240931. doi: 10.1371/journal.pone.0240931 (PMC7577470; doi:10.1371/journal.pone.0240931)
Supplement: S7 Table — Results of generalized linear mixed models of the proportion flooded of survey units among four land cover types in the Central Valley and three regions of central California, USA. Data collected during annual shorebird surveys from 2011 to 2016. Models tested differences in abundance between non-drought years and drought years (2013 to 2015 for drought models; 2014 to 2015 for extreme drought models). Parameter estimates (β) and 95% confidence intervals (CI) are back-transformed to the scale of the response variable. p-values are from a null-hypothesis likelihood-ratio test on the t-statistic for each parameter. Statistically significant p-values are in italics. Deviance scores estimate fit to the data for each model; a lower score represents better fit. (DOCX) [file pone.0240931.s009.docx]

**S7 Table. Results of models of the proportion flooded of survey units in central California, USA**

**.**

Results of generalized linear mixed models of the proportion flooded of survey units among four land cover types in the Central Valley and three regions of central California, USA. Data collected during annual shorebird surveys from 2011 to 2016. Models tested differences in abundance between non-drought years and drought years (2013 to 2015 for drought models; 2014 to 2015 for extreme drought models). Parameter estimates (β) and 95 % confidence intervals (CI) are back-transformed to the scale of the response variable and represent the proportional change in shorebird abundance from non-drought to drought years. *P*-values are from a null-hypothesis likelihood-ratio test on the t-statistic for each parameter. Statistically significant p-values are in italics. Deviance scores estimate fit to the data for each model; a lower score represents better fit.
