# Supplementary material for: Effects of drought on the abundance and distribution of non-breeding shorebirds in central California, USA
Source: PLoS One. 2020 Oct 21;15(10):e0240931. doi: 10.1371/journal.pone.0240931 (PMC7577470; doi:10.1371/journal.pone.0240931)
Supplement: Attachment — Submitted filename: PONE-D-20-20377_RSC.pdf [file pone.0240931.s011.pdf]

PLOS ONE

Effects of drought on the abundance and areas of concentrations of non-breeding shorebirds in central California,

--Manuscript Draft--

Manuscript Number:	PONE-D-20-20377
Article Type:	Research Article
Full Title:	Effects of drought on the abundance and areas of concentrations of non-breeding shorebirds in central California,
Short Title:	Effects of drought on the abundance of non-breeding shorebirds in central California, USA.
Corresponding Author:	Blake Conservation Barbaree<br>Point Blue Conservation Science<br>Petaluma, California UNITED STATES
Keywords:	shorebird ecology; Central Valley; drought; wetlands; shorebird abundance
Abstract:	Conservation of migratory species requires anticipating their response to extreme climatic events, such as severe drought, that can cause changes in abundance and areas of concentration. Responses of highly-mobile shorebirds to drought may vary by geographic location, individual species, and drought severity. We studied the effects of drought on non-breeding shorebirds and their habitats during a multiyear drought using annual surveys between 15 November and 15 December from 2011 to 2016 within interior and coastal areas of central California, USA. In the Central Valley, both habitat availability and the abundance of shorebirds was lower during drought years (2013−2015) when compared to non-drought years. In 2014, we also found evidence of large aggregations of shorebirds near the Sacramento River. Drought effects on abundance were most evident for black-necked stilt in managed wetlands, greater (Tringa melanoleuca) and lesser yellowlegs (T. flavipes) in both wetlands and rice fields, and killdeer (Charadrius vociferous) and long-billed dowitchers (Limnodromus scolopaceus) in all land cover types. On the coast, lower abundance of both yellowlegs and dowitchers during drought years in San Francisco Bay suggested these species have high vulnerability to the effects of drought. Conversely, some species responded to drought by moving to coastal sites; the abundance of dunlin (Calidris alpina) was higher in drought years than in non-drought years at two coastal sites (San Francisco Bay and Point Reyes), and for least sandpiper (C. minutilla) abundance increased at Point Reyes. Our results indicate that water and wildlife conservation investments should be increased and coordinated across interior and coastal regions in central California. We recommend long-term investment in securing wetlands and agricultural lands with reliable water for wildlife, and emergency plans to maximize the amount flooded habitats during years of extreme drought in both interior and coastal regions.
Order of Authors:	Blake A. Barbaree
	Matthew E. Reiter
	Catherine M. Hickey
	Khara M. Strum
	Jennifer E. Isola
	Scott Jennings
	L. Max Tarjan
	Cheryl M. Strong
	Lynne E. Stenzel
	W. David Shuford
Additional Information:	

Powered by Editorial Manager® and ProduXion Manager® from Aries Systems Corporation

Question	Response
**Financial Disclosure**<br><br>Enter a financial disclosure statement that describes the sources of funding for the work included in this submission. Review the submission guidelines for detailed requirements. View published research articles from *PLOS ONE* for specific examples.<br><br>This statement is required for submission and **will appear in the published article** if the submission is accepted. Please make sure it is accurate.<br><br>**Unfunded studies**<br>Enter: *The author(s) received no specific funding for this work.*<br><br>**Funded studies**<br>Enter a statement with the following details:<br>• Initials of the authors who received each award<br>• Grant numbers awarded to each author<br>• The full name of each funder<br>• URL of each funder website<br>• Did the sponsors or funders play any role in the study design, data collection and analysis, decision to publish, or preparation of the manuscript?<br>• **NO** - Include this sentence at the end of your statement: *The funders had no role in study design, data collection and analysis, decision to publish, or preparation of the manuscript.*<br>• **YES** - Specify the role(s) played.<br><br>* typeset	Funding for this research was provided to Point Blue Conservation Science for the Migratory Shorebird Project and Pacific Flyway Shorebird Survey by the David and Lucile Packard Foundation http://packard.org/ and United States Forest Service International Programs https://www.fs.usda.gov/about-agency/international-programs, and to the Migratory Bird Conservation Partnership (Point Blue Conservation Science, Audubon California, The Nature Conservancy) by the S. D. Bechtel, Jr. Foundation http://sdbjrfoundation.org/. The funders had no role in study design, data collection and analysis, decision to publish, or preparation of the manuscript.
**Competing Interests**<br><br>Use the instructions below to enter a competing interest statement for this submission. On behalf of all authors, disclose any competing interests that could be perceived to bias this work—acknowledging all financial support and any other relevant financial or non-financial competing interests.<br><br>This statement **will appear in the published article** if the submission is	The authors have declared that no competing interests exist.

accepted. Please make sure it is accurate. View published research articles from *PLOS ONE* for specific examples.<br><br>**NO authors have competing interests**<br><br>Enter: *The authors have declared that no competing interests exist.*<br><br>**Authors with competing interests**<br><br>Enter competing interest details beginning with this statement:<br><br>*I have read the journal's policy and the authors of this manuscript have the following competing interests: [insert competing interests here]*<br><br>* typeset	
**Ethics Statement**<br><br>Enter an ethics statement for this submission. This statement is required if the study involved:<br><br>• Human participants<br>• Human specimens or tissue<br>• Vertebrate animals or cephalopods<br>• Vertebrate embryos or tissues<br>• Field research<br><br>Write "N/A" if the submission does not require an ethics statement.<br><br>General guidance is provided below. Consult the submission guidelines for detailed instructions. **Make sure that all information entered here is included in the Methods section of the manuscript.**	N/A

Powered by Editorial Manager® and ProduXion Manager® from Aries Systems Corporation

**Format for specific study types**<br><br>**Human Subject Research (involving human participants and/or tissue)**<br>• Give the name of the institutional review board or ethics committee that approved the study<br>• Include the approval number and/or a statement indicating approval of this research<br>• Indicate the form of consent obtained (written/oral) or the reason that consent was not obtained (e.g. the data were analyzed anonymously)<br><br>**Animal Research (involving vertebrate animals, embryos or tissues)**<br>• Provide the name of the Institutional Animal Care and Use Committee (IACUC) or other relevant ethics board that reviewed the study protocol, and indicate whether they approved this research or granted a formal waiver of ethical approval<br>• Include an approval number if one was obtained<br>• If the study involved *non-human primates*, add *additional details* about animal welfare and steps taken to ameliorate suffering<br>• If anesthesia, euthanasia, or any kind of animal sacrifice is part of the study, include briefly which substances and/or methods were applied<br><br>**Field Research**<br><br>Include the following details if this study involves the collection of plant, animal, or other materials from a natural setting:<br>• Field permit number<br>• Name of the institution or relevant body that granted permission	
**Data Availability**<br><br>Authors are required to make all data underlying the findings described fully available, without restriction, and from the time of publication. PLOS allows rare exceptions to address legal and ethical concerns. See the PLOS Data Policy and FAQ for detailed information.	No - some restrictions will apply

A Data Availability Statement describing where the data can be found is required at submission. Your answers to this question constitute the Data Availability Statement and **will be published in the article**, if accepted.<br><br>**Important:** Stating 'data available on request from the author' is not sufficient. If your data are only available upon request, select 'No' for the first question and explain your exceptional situation in the text box.<br><br>Do the authors confirm that all data underlying the findings described in their manuscript are fully available without restriction?	
**Describe where the data may be found in full sentences. If you are copying our sample text, replace any instances of XXX with the appropriate details.**<br><br>• If the data are **held or will be held in a public repository**, include URLs, accession numbers or DOIs. If this information will only be available after acceptance, indicate this by ticking the box below. For example: *All XXX files are available from the XXX database (accession number(s) XXX, XXX.).*<br>• If the data are all contained **within the manuscript and/or Supporting Information files**, enter the following: *All relevant data are within the manuscript and its Supporting Information files.*<br>• If neither of these applies but you are able to provide **details of access elsewhere**, with or without limitations, please do so. For example:<br><br>*Data cannot be shared publicly because of [XXX]. Data are available from the XXX Institutional Data Access / Ethics Committee (contact via XXX) for researchers who meet the criteria for access to confidential data.*<br><br>*The data underlying the results presented in the study are available from (include the name of the third party*	The data cannot be shared publicly in its entirety. The data were collected under multiple data sharing agreements that permit various levels of access by the public. The data are stored within a repository named the California Avian Data Center https://data.prbo.org/cadc2/, which is a node of the Avian Knowledge Network. The data can be previewed using an custom webtool found under the "Explore Data" tab on the website for the Pacific Flyway Shorebird Survey http://data.prbo.org/apps/pfss/<br><br>Here we choose a conservative approach to limit access to the data used in the study. However, we are currently evaluating the possibility of modifying the dataset to allow inclusion as supplemenal information without breaking any of the multiple data sharing agreements that apply to the data.

Powered by Editorial Manager® and ProduXion Manager® from Aries Systems Corporation

and contact information or URL).<br>• This text is appropriate if the data are owned by a third party and authors do not have permission to share the data.<br><br>* typeset	
Additional data availability information:	

Manuscript

Click here to access/download;Manuscript;Barbaree et al_Shorebirds and drought in central CA.docx

Effects of drought on the abundance and areas of concentrations of non-breeding shorebirds in central California, USA.

Blake A. Barbaree[1]*, Matthew E. Reiter[1], Catherine M. Hickey[1], Khara M. Strum[2], Jennifer E. Isola[3], Scott Jennings[4], L. Max Tarjan[5], Cheryl M. Strong[6], Lynne E. Stenzel[1], W. David Shuford[1]

[1] Point Blue Conservation Science, 3820 Cypress Drive #11, Petaluma, California, United States of America.

[2] Audubon California, 455 Capitol Mall, Suite 415, Sacramento, California, United States of America.

[3] United States Fish and Wildlife Service, Sacramento National Wildlife Refuge Complex, 752 Country Road 99W, Willows, California, United States of America.

[4] Audubon Canyon Ranch, PO Box 808, Marshall, California, United States of America.

[5] San Francisco Bay Bird Observatory, 524 Valley Way, Milpitas, California, United States of America.

[6] United States Fish and Wildlife Service, Newport Field Office, 2127 SE Marine Science Drive Newport, Oregon, United States of America.

* Corresponding author

E-mail: bbarbaree@pointblue.org

Keywords: shorebird ecology; Central Valley; drought; wetlands; shorebird abundance.

Abstract

Conservation of migratory species requires anticipating their response to extreme climatic events, such as severe drought, that can cause changes in abundance and areas of concentration. Responses of highly-mobile shorebirds to drought may vary by geographic location, individual species, and drought severity. We studied the effects of drought on non-breeding shorebirds and their habitats during a multiyear drought using annual surveys between 15 November and 15 December from 2011 to 2016 within interior and coastal areas of central California, USA. In the Central Valley, both habitat availability and the abundance of shorebirds was lower during drought years (2013−2015) when compared to non-drought years. In 2014, we also found evidence of large aggregations of shorebirds near the Sacramento River. Drought effects on abundance were most evident for black-necked stilt in managed wetlands, greater (*Tringa melanoleuca*) and lesser yellowlegs (*T. flavipes*) in both wetlands and rice fields, and killdeer (*Charadrius vociferous*) and long-billed dowitchers (*Limnodromus scolopaceus*) in all land cover types. On the coast, lower abundance of both yellowlegs and dowitchers during drought years in San Francisco Bay suggested these species have high vulnerability to the effects of drought. Conversely, some species responded to drought by moving to coastal sites; the abundance of dunlin (*Calidris alpina*) was higher in drought years than in non-drought years at two coastal sites (San Francisco Bay and Point Reyes), and for least sandpiper (*C. minutilla*) abundance increased at Point Reyes. Our results indicate that water and wildlife conservation investments should be increased and coordinated across interior and coastal regions in central California. We recommend long-term investment in securing wetlands and agricultural lands with reliable water for wildlife, and emergency plans to maximize the amount flooded habitats during years of extreme drought in both interior and coastal regions.

Introduction

Wildlife populations are vulnerable to extreme climatic events such as severe drought, and many climate projections suggest that future droughts will likely be longer and drier in many regions [1,2]. Extreme drought results in resource scarcity, and the resulting impacts on wildlife must be considered when developing effective conservation strategies for freshwater habitats and wildlife [3,4]. Evaluating when and where the effects of drought result in resource scarcity, and the resulting influence on wildlife populations, requires long-term monitoring [5]. In the case of highly-mobile species such as migratory birds, long-term monitoring must also occur over a broad scale.

Drought has widespread impacts on avian communities [6,7], especially those in regions dominated by freshwater ecosystems [8]. Moreover, extensive habitat loss, land modification, and human use of fresh water compounds the impacts of drought on migratory birds that are reliant on freshwater habitats within interior regions [9]. A recent multiyear drought in western North America was the driest period in more than 1200 years in California [10], which led to shortages of fresh water for both humans and wildlife from 2013 to 2016 [11,12]. In California's Central Valley, where < 10% of historic wetlands remain [13], scarcity of fresh water during the drought likely caused food and energy shortfalls for waterfowl [14]. Like waterfowl, nonbreeding shorebird populations in the northern Central Valley rely on managed wetlands [15], and post-harvest winter-flooded rice fields [16]. However, the effects of drought on populations of non-breeding shorebirds remains poorly understood.

The response of avian communities to the suppression of key resources by drought depends on their habitat preferences and the magnitude of the drought [17]. As a drought intensifies over time, certain regions and habitat types will likely be more affected during early

stages of a drought, whereas impacts to other regions or habitats may only be evident once the drought reaches an extreme level. In the Central Valley, some shorebird species are known to respond to a seasonal reduction in flooded agricultural lands, primarily within northern areas from February to April, by moving to areas with more stable habitat availability within the Central Valley and nearby coastal regions [18] or by shifting local habitat associations [19]. If shorebirds respond similarly to multiyear droughts that limits habitat for the duration of the non-breeding season, shorebird abundance and areas of concentrations could change significantly for an extended period in the Central Valley. Understanding how shorebirds respond to drought is needed to formulate effective conservation plans for fresh water and wildlife because there is the potential for escalating competition between humans and wildlife for limited freshwater resources if a warmer, drier climate persists [20].

In this study, we used annual surveys of non-breeding shorebirds at sites in the Central Valley and nearby coastal sites to characterize the response of shorebirds to drought, and document patterns of habitat availability from 2011 to 2016. We used two classifications of drought severity for the multiyear drought, a 3-year drought from 2013 to 2015 and a 2-year extreme drought from 2014 to 2015 (see Methods for more details), to elucidate the magnitude and timing of effects during the drought. Our primary hypothesis was that less surface water (thus lower habitat availability) in the Central Valley during drought years would lead to lower abundance of all shorebird species combined when compared to non-drought years.

We relied on past research to generate predictions for individual shorebird species that would decline in abundance during drought years within managed wetlands or agricultural lands, and those that may shift areas of concentration from the Central Valley to coastal sites. Thus, we predicted lower abundance during drought years than in non-drought years for black-necked stilt

(*Himantopus mexicanus*) in managed wetlands, and for killdeer (*Charadrius vociferous*), greater yellowlegs (*Tringa melanoleuca*), and dunlin (*Calidris alpina*) in agricultural lands (primarily rice fields) [21]. Based on the responses of shorebirds to changing distributions of surface water in the Central Valley [18], we also predicted that areas of shorebird concentrations would shift northward away from the driest, southern areas in the Central Valley, and to nearby coastal regions, where habitat availability is mostly related to tidal cycles and remains relatively stable even during extreme drought. Thus, we expected higher abundance of black-necked stilt [22], dunlin [18], and long-billed dowitcher (*Limnodromus scolopaceus*) [23] in the San Francisco Bay estuary during drought years because they have shown evidence of movements between interior and coastal sites. In estuaries of the Point Reyes peninsula, we expected higher abundance of killdeer, dunlin, and least sandpiper (*Calidris minutilla*), during drought years [21,24].

Study area

The western portion of central California includes several regions of importance for shorebirds in the Pacific Flyway [25]. The Central Valley of California is an interior, low elevation basin of approximately 52,000 (km^2) between the Sierra Nevada and Coast Ranges and supports over 1 million shorebirds each year [21]. It extends 642 km from north to south and 48 to 112 km from east to west. Runoff from the surrounding montane watersheds is the primary source of fresh water that supports habitat for migratory shorebirds, and water supplies are subject to human management decisions in response to annual and multiyear variation in precipitation. Annual and seasonal precipitation is highly variable ranging from as little as 50% to more than 300% of the long-term averages [26] with most precipitation traditionally occurring

from November to March. The climate is progressively drier from north to south, and land use is primarily agriculture with expanding urban areas that are concentrated near the convergence of the Sacramento and San Joaquin rivers. We defined four sub-regions in the Central Valley: Sacramento Valley, Yolo-Delta, San Joaquin, and Tulare (Fig 1); these sub-regional boundaries were used from 2006-2015 by the Central Valley Joint Venture (https://www.centralvalleyjointventure.org/). The sub-regional boundaries used in this study were updated by the Central Valley Joint Venture in 2016, but this change did not affect the sub-regional affiliation of any survey unit in our study.

The coastal regions of our study area included the San Francisco Bay estuary (hereafter San Francisco Bay) and a set of smaller estuaries along the Point Reyes peninsula (Fig 1). San Francisco Bay is the largest estuary on the Pacific coast of North America and centered around 75 km west of the Yolo-Delta sub-region in the Central Valley. San Francisco Bay is used by over half a million shorebirds during migration and winter; see [27] for details on the features and shorebird use of San Francisco Bay. We consider Suisun Marsh, which lies west of the Yolo-Delta sub-region and the convergence of the Sacramento and San Joaquin rivers, as part of San Francisco Bay, primarily because the managed wetlands of Suisun Marsh do not rely primarily on deliveries of fresh water for flooding and lack surrounding agricultural lands, distinguishing it from similar wetlands in the Central Valley.

The Point Reyes peninsula, on the outer coast ~30 km northwest of San Francisco Bay, includes three smaller, distinct estuaries—Tomales Bay, Bolinas Lagoon, and Drakes-Limantour estuary system (including Drakes and Limantour Esteros)—and Abbotts Lagoon, a true lagoon. For details on the features and shorebird use of the estuaries of Point Reyes see [28,29,30].

Fig 1. Study area for annual surveys of non-breeding shorebirds from 2011 to 2016 in California, USA. Labels are included for Central Valley sub-regions and coastal regions. Shaded relief shows elevation as <150m (white) to >1800 m (darkest gray). Land cover types derived primarily from satellite imagery; see Barbaree et al. 2018) for more details. Other suitable habitats included grasslands/pasture, corn fields, other irrigated row or field crops, freshwater lakes/ponds, and wastewater treatment ponds. Area of all survey units has been enlarged to identify locations of surveys for the purposes of this map.

Methods

We used data collected annually from 2011 to 2016, for the Pacific Flyway Shorebird Survey (pointblue.org/pfss), a multi-scale survey conducted by biologists and volunteers trained to use a standardized survey protocol and coordinated via a network of partner organizations. Surveys occurred from 15 November to 15 December, when most shorebird migration has ceased in California [18] and there is controlled flooding of managed wetlands and some post-harvest agricultural lands in the Central Valley [12,31]. While surveys could occur anytime during the survey period, we scheduled surveys on the same or consecutive days within large complexes of flooded habitats in the Central Valley and within portions of each coastal region unless constrained by access to sites; surveyed were postponed if potentially constrained adverse viewing conditions (i.e., weather), access, or availability of surveyors. Surveys of coastal sites occurred at a tidal stage that was standardized across years within each estuary. Surveys occurred across a high tide in San Francisco Bay, and during a rising tide in each Point Reyes estuary. Each unit was surveyed for a minimum of two minutes or until all individuals had been counted.

To characterize habitat availability, we estimated the proportional area of surface water within each survey unit (hereafter flooded portion) and recorded the dominant land cover type.

For each defined survey unit, we counted the number of shorebirds (suborders Scolopaci and Charadrii) on the ground. We attempted to identify all individuals to species, but when this was not possible, we used a proportional allocation method that we modified from previous work [25] to correct the totals for species that would have been undercounted because of their inclusion in unidentified, mixed species (UMS) flocks. In UMS flocks, we counted the total flock size and identified candidate species (least sandpiper, western sandpiper, and dunlin) in the field. We later estimated the proportional composition of candidate species in the flocks based of the composition of those species that were identified in the survey unit and within the region or sub-region where the flock occurred. Thus, the total for species *i* was c_i, which included the total identified and counted in the field, added to e_i, the estimated number included in UMS flocks of size U individuals: $e_i = f_i / (f_i + f_j + \ldots) * U$, where $(f_i + f_j + \ldots)$ is the total count of candidate species identified in the field. We allocated counts to western sandpiper where appropriate, but did not analyze data for this species because of relatively low abundance in the Central Valley [21] when compared to nearby San Francisco Bay [27].

Survey design

In the Central Valley, we conducted 7,607 surveys of 1,556 individual survey units from 2011 to 2016. Of the 1,856.44 ± 146.40 km^2 surveyed annually in this region, the Sacramento Valley accounted for 80%, Tulare 8%, San Joaquin 7%, and Yolo-Delta 7% (S1 Table). We surveyed 58 routes on public and private roads and within complexes of managed wetland impoundments. We identified 40 survey routes using a random selection process and placed 18

within wetlands of federal or state refuges. We began our random selection process by refining the sampling frame to include only areas with high potential for use by shorebirds [32]. Next, a retrospective analysis using satellite imagery of surface water within shorebird habitats excluded areas that were flooded from November to January in <30% of years 2000 - 2010, which removed approximately 90% of the Central Valley. Forty township grids (9.66 x 9.66 km; Public Land Survey System; http://www.nationalatlas.gov) were then selected to contain a single survey route using a systematic random process [33]. Grids selected for survey routes in the Sacramento Valley and Yolo-Delta sub-regions had ≥50% of 30 x 30 m cells classified as rice fields (*n* = 35 grids). Grids selected in the San Joaquin sub-region had ≥50% of cells classified as managed wetlands (*n* = 5). We did not randomly select routes in the Tulare sub-region. The remaining 18 routes were within federal or state refuges from the Sacramento National Wildlife Refuge (NWR) Complex in the Sacramento Valley sub-region south to Kern and Pixley NWRs in the Tulare sub-region, except for the Cosumnes River preserve managed by the Nature Conservancy in the Yolo-Delta sub-region. Each randomly selected route consisted of 14 – 20 survey units.

In the Central Valley, we used three types of fixed spatial boundaries to delineate survey units: fixed-radius point counts (*n* = 774), restricted area searches (*n* = 409), and complete area searches (*n* = 377). Fixed radius (0.16 km) point counts were used only for randomly-selected road routes and spaced approximately every 0.8 km along routes that were up to 23 km in length on publicly accessible roads. We used restricted area searches for seasonally flooded impoundments on federal, state, or private lands; survey unit boundaries were located a fixed distance (0.16 km) from roads and bounded on the sides by levees that defined each wetland management unit. We used complete area searches for comprehensive counts of individual seasonally flooded impoundments within federal refuges; survey unit boundaries were defined

entirely by levee boundaries of individual impoundments, which varied in size. Complete area searches were conducted by U.S. Fish and Wildlife Service personnel exclusively within the Sacramento NWR and Kern-Pixley NWR complexes.

In coastal regions, we surveyed primarily within tidally-influenced wetlands, intertidal mudflats, tidal salt marsh, managed ponds, salt production ponds and other associated or nearby managed impoundments. We designed boundaries of coastal survey units using coastline boundaries, habitat delineations, levees, and a maximum distance from the coastline of 300 m. In San Francisco Bay, we subsampled shorebird habitats using a stratified random process that weighted survey units using the natural logarithm of total shorebirds counted during comprehensive surveys from 2006 to 2008 [34]. This resulted in 1,095 surveys (n = 258 survey units) with an annual mean area of 1,570.64 ± 60.77 km^2 surveyed (S1 Table); there was no survey in Suisun Marsh during 2011 and some portions of San Francisco Bay were not accessible in 2014. In Point Reyes, we conducted comprehensive surveys of each estuary using boundaries that aligned with pre-existing survey units in Bolinas Lagoon [30], Tomales Bay [29], and Limantour Estero, an arm of Drakes Estero [28]. In 2013 we added additional survey units for Abbotts Lagoon and intertidal habitats within the remainder of Drakes Estero. This resulted in 330 surveys of 64 survey units with an annual mean of 149.28 ± 24.72 km^2 surveyed in Point Reyes.

Dominant land cover types in the Central Valley

We categorized individual survey units in the Central Valley into four land cover types: managed wetlands (58% of total area surveyed annually), rice fields (19%), other suitable habitats (21%), and miscellaneous (2%; S2 Table). Managed wetlands (wetlands) and post-

harvest rice fields (rice fields) are important targets for conservation actions and account for the majority of potential shorebird habitat that is consistently flooded at depths and durations that benefit shorebirds in the Central Valley [21,31]. We defined "other suitable habitats" as habitats other than wetlands and rice fields that had potential for use by shorebirds when flooded, including grasslands/pasture, corn fields, other irrigated row or field crops, freshwater lakes/ponds, and wastewater treatment ponds; we did not sample any individual other suitable habitat extensively. We defined "miscellaneous" land cover types as having low potential for use by shorebirds, and included developed, forested, riverine, orchard, and vineyard.

We identified a single dominant cover type for each survey unit using a combination of satellite imagery and data collected during field surveys. We began by using a spatial data layer depicting land cover in the Central Valley (30 x 30 m pixels) [18]; the dominant cover type was the one with the highest proportion of land cover within each survey unit boundary. We used the estimated cover type from GIS data when it was consistent with field observations or when the latter were not available. When the GIS-estimated cover type differed from the observed cover type, we manually assigned a cover type by visually assessing satellite imagery of the unit from recent years in Google Earth Engine (explorer.earthengine.google.com). Cover types for individual units remained static across years except for eight units for which both an observer and satellite imagery documented a change in cover type.

Focal species

In the Central Valley, we investigated the effects of drought on the abundance of all shorebirds species and six focal species. In coastal regions, we investigated only focal species because the geographic distribution of some shorebird species is primarily on the coast (e.g.,

western sandpiper; *Calidris mauri*). All focal species commonly overwintered within both interior and coastal wetlands of central California. We identified the killdeer, black-necked stilt, greater yellowlegs, dunlin, least sandpiper, and long-billed dowitcher as focal species because of their relative abundance and occurrence during November and December in the Central Valley and coastal California [21,25,30]. We combined counts of greater yellowlegs and lesser yellowlegs (*Tringa flavipes*; hereafter yellowlegs) as well as long-billed dowitcher and short-billed dowitcher (*Limnodromus griseus*; hereafter dowitchers) because they often could not be distinguished during surveys. However, for dowitchers, we considered our results to be applicable to only long-billed dowitchers because they likely accounted for most, if not all, of dowitchers encountered in the Central Valley [21], and evidence that long-billed dowitchers accounted for most dowitchers in San Francisco Bay during the wintering period [35]. Other species that we encountered in the Central Valley included: black-bellied plover (*Pluvialis squatarola*), snowy plover (*Charadrius nivosus*), American avocet (*Recurvirostra americana*), long-billed curlew (*Numenius americanus*), willet (*Tringa semipalmata*), marbled godwit (*Limosa fedoa*), western sandpiper (*C. mauri*), Wilson's phalarope (*Phalaropus tricolor*), and Wilson's snipe (*Gallinago delicata*).

Data summary and analysis

In the Central Valley, we summed counts of all shorebird species (hereafter total shorebirds) and also grouped total shorebird counts by sub-region and land cover type, whereas for counts of individual focal species and estimates of the flooded portion of survey units, we grouped observations by land cover type only. In San Francisco Bay, we analyzed counts of focal species and estimates of flooded portion of survey units. In Point Reyes, we analyzed

counts of focal species, and we did not analyze data on flooding of survey units because of a paucity of data at most sites during 2011 and 2012. To qualitatively assess shifts of areas of concentration of shorebirds in the Central Valley, we calculated a standardized mean density from 2011 to 2016 and then mapped the relative annual mean density (count/survey area in km^2) of total shorebirds by sub-region. For subsequent statistical analyses of shorebird abundance, we modelled abundance (count) of total shorebirds in the Central Valley and abundance of focal shorebirds in the Central Valley and coastal regions; see S1-S5 Tables for mean annual total counts and annual density estimates presented as a proxy for abundance in each region, sub-region, and land cover type.

To test for the effects of drought on both annual abundance and habitat availability (flooded portion of survey units), we used two separate generalized linear mixed effects models that tested for differences in abundance (birds counted) during drought and non-drought years. We identified 2013 to 2015 as drought years based on the water year type as defined by the California Department of Water Resources for the Sacramento and San Joaquin River Valleys based on the projected runoff (million acre feet) on 1 May (see http://cdec.water.ca.gov/cgi-progs/iodir/WSIHIST for details on the level for each classification and data access) [12]. Over the six years of the study, the Sacramento Valley was classified as drought or critical from 2013 to 2015 and below normal (2012, 2016) or wet (2011) in other years. The San Joaquin Valley was classified as drought or critical in all years except 2011. For the full Central Valley, we considered a drought year to include any water year designated as “drought” or “critical” in both the Sacramento and San Joaquin Valleys. We then analyzed both 3-year drought (2013–2015) and 2-year extreme drought (2014–2015) periods; we defined an extreme drought year as having at least one drought year immediately preceding it.

We used a negative binomial distribution with a “log” link for models of shorebird abundance. All models were fitted using maximum likelihood estimation and included a single fixed factor (abundance or flooded portion) and a random effect term to account for repeated surveys at individual survey units. Models of abundance included an offset term of the natural log of area (km^2) of each survey unit to control for variation in the size of survey units. We used a null-hypothesis likelihood ratio test on the *t*-statistic for each parameter to test for significance; we considered $p \leq 0.05$ to represent moderate to convincing evidence for a difference in response variables between non-drought and drought years, and $p = 0.06 – 0.10$ to provide suggestive but inconclusive evidence of such a difference [36,37]. For negative binomial models, we report coefficients (β) after back transforming them to the scale of the response variable (with the inverse of the link function); thus reported β represent the proportional change in shorebird abundance from non-drought to drought years. All analyses were conducted in Program R (R Core Team, Version 3.5.1; http://www.r-project.org/) and package glmmTMB [38].

Results

Patterns of shorebird use in the interior

Despite wetlands accounting for the majority of area surveyed in the Central Valley (58%; S2 Table), only two of six focal species had a majority of individuals counted within wetlands. For killdeer, 59% of 4,994 individuals were in rice fields, 24% in wetlands, 12% in other suitable habitats, and 5% in miscellaneous cover types; killdeer also accounted for 80% of the 355 individual shorebirds counted within miscellaneous. For black-necked stilt, 89% of 11,982 individuals were in wetlands, 6% in rice fields, 5% in other suitable habitats, and 0% in

miscellaneous. For yellowlegs, 52% of 2,387 individuals were in wetlands, 43% in rice fields, 4% in other suitable habitats, and <1% in miscellaneous. For dunlin, 71% of 20,502 individuals were in rice fields, 26% in wetlands, 3% in other suitable habitats, and 0% in miscellaneous. For least sandpiper, 55% of 16,724 individuals were in rice fields, 36% in wetlands, 9% in other suitable habitats, and <1% in miscellaneous. For dowitchers, 55% of 28,735 individuals were in rice fields, 41% in wetlands, 5% in other suitable habitats, and <1% in miscellaneous (S3 Table). We counted comparatively few individuals of uncommon to rare non-focal shorebird species, particularly during 2015 and 2016 (S4 Table).

Patterns of relative mean density of total shorebirds in the Central Valley varied annually and among sub-regions (Fig 2). In the Sacramento Valley and Yolo-Delta, shorebird density was considerably higher than average during 2012 and 2014, and lower than average during 2013 and 2015. In 2014, higher than average shorebird density in northern sub-regions was likely related to three of the five highest counts of total shorebirds, which consisted primarily of dunlin, occurring in survey units of rice fields near the Sacramento River; of the twenty highest counts of total shorebirds, 5% occurred in 2013, 25% in 2014 (all near the Sacramento River), and 5% in 2015. San Joaquin was the only sub-region where annual density of total shorebirds was near average in 2013; no survey occurred at the Los Banos Wildlife Area in 2014, which may have contributed to the low relative mean density and less flooding of survey units in the sub-region (S2 Figure). In the Tulare sub-region, annual density declined rapidly from 2012 to 2013 and then remained low. Notably, no sub-region had higher than average shorebird density in the non-drought year of 2016. Across all sub-regions in the Central Valley, shorebird density was highest during 2012 (0.0012 birds per km^2), which was a year of below normal runoff following an

extremely wet year, and more than twice the density observed during 2015 (0.0005; S1 Table), which was the third of three consecutive years of drought.

Fig 2. Relative mean density of total shorebirds (birds per km^2) by sub-region from 2011 to 2016 in the Central Valley, California, USA. The density categories show the proportional difference between the annual mean estimate of density and the standardized mean density from 2011 to 2016 in each sub-region; a relative mean density of 1.0 is equal to the standardized mean annual density (birds per km^2).

Model estimates of total shorebird abundance in all cover types across the Central Valley provided moderate to convincing evidence of lower abundance during both the periods of 3-year drought (β = 0.78, 95% CI: 0.63, 0.98, p = 0.03) and 2-year extreme drought than in non-drought years (β = 0.71, 95% CI: 0.56, 0.91, p = 0.007; Fig 3). Among models of total shorebirds by cover type, we found no differences in abundance during the periods of 3-year drought or 2-year extreme drought than in non-drought years within wetlands (β = 0.89, 95% CI: 0.65, 1.23, p = 0.14; β = 0.78, 95% CI: 0.54, 1.11, p = 0.17), rice fields (β = 0.78, 95% CI: 0.55, 1.09, p = 0.49; β = 0.75, 95% CI: 0.52, 1.09, p = 0.14), or other suitable habitats (β = 0.74, 95% CI: 0.32, 1.68, p = 0.47; β = 0.64, 95% CI: 0.26, 1.60, p = 0.34); however, in miscellaneous cover types, we found significantly lower abundance of total shorebirds during both the periods of 3-year drought (β = 0.15, 95% CI: 0.04, 0.55, p = 0.004) and 2-year extreme drought than in non-drought years (β = 0.10, 95% CI: 0.02, 0.45, p = 0.003). Among models of focal species, only killdeer showed evidence of lower abundance across the Central Valley during both the periods of 3-year drought and 2-year extreme drought than in non-drought years (Fig 4 and S6 Table). For dowitchers, we found moderate evidence for lower abundance across the Central Valley during the 3-year period

of drought compared to non-drought years, and somewhat suggestive evidence for lower abundance during 2-year period of extreme drought.

Fig 3. Estimated abundance of total shorebirds during a drought year among four dominant cover types. Results from generalized linear mixed models of the annual abundance of total shorebirds from 2011 to 2016 in the Central Valley, California, USA. Plots for each land cover type show the estimated proportional change in total shorebird abundance relative to a non-drought year from two models; drought models classified drought years as a period of a 3-year drought (2013–2015) or 2-year extreme drought (2014–2015). The solid black line represents no change from the estimated abundance of total shorebirds during a non-drought year. We defined “other suitable habitats” as those with potential for use by shorebirds when flooded, including grasslands/pasture, corn fields, other irrigated row or field crops, freshwater lakes/ponds, and wastewater treatment ponds. We defined “miscellaneous” land cover types as having low potential for use by shorebirds, and included developed, forested, riverine, orchard, and vineyard.

Fig 4. Estimated abundance for six focal species during a drought year in central California, USA. Results from generalized linear mixed models of the annual abundance of six shorebirds from 2011 to 2016 in three regions of central California, USA. Plots for each region show the estimated proportional change in total shorebird abundance relative to a non-drought year from two models; drought models classified drought years as a period of a 3-year drought (2013–2015) or 2-year extreme drought (2014–2015). The solid black line represents no change from the estimated abundance of total shorebirds during a non-drought year.

For models of abundance within wetlands in the Central Valley, only for the black-necked stilt did we find moderate evidence of effects of drought in both drought periods (Table

1). We found moderate evidence of a lower abundance for dowitchers over the period of 3-year drought than in non-drought years, and suggestive evidence of lower abundance during the period of 2-year extreme drought for least sandpiper. In rice fields, we found moderate evidence of lower abundance of the killdeer in both drought periods when compared to non-drought years; models also indicated suggestive evidence of lower abundance of yellowlegs during the period of 2-year extreme drought, and of dowitchers for the period of 3-year drought. Within other suitable habitats, we found convincing evidence of lower abundance of the dunlin and least sandpiper during the period of 3-year drought than in non-drought years, and the size of the estimated effect for both species showed abundance during the period of 3-year drought was less than 1% of non-drought abundance. We found moderate evidence for an effect of 3-year drought on the abundance of dowitchers in other suitable habitats, but in contrast to our prediction, they increased in abundance during drought years compared to non-drought years.

Table 1. Results of models of shorebird abundance among cover types in the Central Valley, California, USA.

Focal species	Model	Wetlands				Rice fields				Other suitable habitats			
		Deviance	β	95% CI	*p*-value	Deviance	β	95% CI	*p*-value	Deviance	β	95% CI	*p*-value
Killdeer	drought	3,443.6	1.05	0.74 1.48	0.79	5,092.3	0.69	0.33 1.41	*0.03*	1,048.4	0.55	0.23 1.35	0.20
	extreme drought	3,443.5	0.83	0.54 1.26	0.38	5,090.4	0.67	0.49 0.91	*0.01*	1,048.8	0.56	0.21 1.51	0.25
Black-necked stilt	drought	6,998.6	0.68	0.48 0.98	*0.04*	769.7	1.26	0.33 4.86	0.74	401.4	0.90	0.15 5.36	0.91
	extreme drought	6,997.9	0.62	0.41 0.95	*0.03*	769.8	0.96	0.22 4.12	0.95	401.4	0.98	0.13 7.60	0.98
Greater & Lesser yellowlegs	drought	2,934.4	0.89	0.60 1.30	*0.54*	2,374.5	0.77	0.47 1.25	0.29	285.8	2.36	0.49 11.31	0.29
	extreme drought	2,934.3	1.15	0.76 1.74	0.51	2,372.8	0.63	0.36 1.07	*0.09*	327.1	1.54	0.30 7.99	0.61
Dunlin	drought	2,174.1	0.78	0.34 1.77	0.55	2,550.9	1.02	0.44 2.34	0.97	222.9	<0.001	<0.001 0.045	*0.003*
	extreme drought	2,174.0	0.72	0.30 1.76	0.47	2,550.0	1.53	0.63 3.73	0.35	223.5	0.27	0.01 12.81	0.51
Least sandpiper	drought	2,619.9	0.74	0.37 1.46	0.38	2,443.1	1.06	0.41 2.73	0.91	355.0	0.001	<0.001 0.059	*0.002*
	extreme drought	2,617.5	0.49	0.23 1.03	*0.06*	2,443.1	0.95	0.34 2.62	0.92	345.5	2.70	0.16 46.17	0.49
Long- & Short-billed dowitcher	drought	3,670.8	0.54	0.28 1.05	*0.07*	1,872.4	0.32	0.08 1.19	*0.09*	291.3	13.44	1.15 157.59	*0.04*
	extreme drought	3,673.1	0.69	0.33 1.41	0.31	1,873.4	0.40	0.12 1.40	0.15	294.0	1.78	0.03 124.84	0.79

Results of generalized linear mixed models of abundance of six focal shorebirds in three dominant land cover types used by shorebirds in the Central Valley. Data collected during annual shorebird surveys from 2011 to 2016. Models tested differences in abundance between non-drought years and drought years (2013 to 2015 for drought models; 2014 to 2015 for extreme drought models).

Parameter estimates (β) and 95 % confidence intervals (CI) are back-transformed to the scale of the response variable. *P*-values are from a null-hypothesis likelihood-ratio test on the *t*-statistic for each parameter. Statistically significant *p*-values are in italics. Deviance scores estimate the goodness of fit for each model; a lower score represents better goodness of fit.

Coastal abundance

In San Francisco Bay, annual density estimates of focal species peaked from 2014 to 2016, including maximum annual estimates for the black-necked stilt in 2014, and for the dunlin and dowitchers in 2015 (S5 Table). In Point Reyes, we recorded zero detections of black-necked stilt and few dowitchers when compared to other regions. Models of abundance of focal species indicated mixed responses among species to drought in San Francisco Bay, and only higher or no difference in abundance in Point Reyes (Fig 4 and S6 Table). We found no difference in killdeer abundance during either drought period when compared to non-drought years in San Francisco Bay or Point Reyes. For the black-necked stilt, we found no difference in abundance during either drought period when compared to non-drought years in San Francisco Bay. For yellowlegs, we found moderate evidence for lower abundance in San Francisco Bay during the period of 2-year extreme drought than in non-drought years ($p = 0.04$). For the dunlin, we found convincing evidence in San Francisco Bay for an increase in abundance during the periods of 3-year drought ($p < 0.001$) and 2-year extreme drought compared to non-drought years ($p = 0.005$), and moderate evidence in Point Reyes during the 3-year period of drought ($p = 0.02$). For the least sandpiper, we found suggestive evidence for an increase in abundance during the 3-year period of drought when compared to non-drought years in Point Reyes ($p = 0.06$). For dowitchers, we found suggestive evidence for lower abundance during the 2-year period of extreme drought when compared to non-drought years in San Francisco Bay ($p = 0.095$).

Habitat availability

In the Central Valley, the mean flooded portion of survey units was highest in 2012 (0.54), declined each year of the drought until the lowest estimate in 2015 (0.39), and then flooding in 2016 was similar to 2011 (0.48; S1 Figure). Annual estimates of the mean flooded portion of survey units by sub-region indicated that the Yolo-Delta and San Joaquin sub-regions had less variable habitat availability from 2011 to 2016 than the Sacramento Valley and Tulare sub-regions (S2 Figure). Models provided convincing evidence of less flooding of survey units during both the periods of 3-year drought and 2-year extreme drought when compared to non-drought years (Fig 5; S7 Table). Among cover types, we found convincing evidence of a less flooding of survey units during both the periods of 3-year drought and 2-year extreme drought within wetlands, rice fields, and other suitable habitats (all $p < 0.001$); there were no differences in the flooded portion of survey units for miscellaneous cover types (both periods of drought $p > 0.78$). In San Francisco Bay, we found no difference in the flooding of survey units during the period of 3-year drought when compared to non-drought years, and a moderate effect of increased flooding during the period of 2-year extreme drought ($p = 0.02$), which was possibly related to habitat restoration in some units and variation in the maximum tidal height during the survey each year.

Fig 5. Estimated proportional change in flooding of survey units during drought years. Results from generalized linear mixed models of the flooded portion of shorebird survey units from 2011 to 2016 in the Central Valley California, USA. Plots for each land cover type show the estimated change in the annual flooded portion of survey units from two models; drought models classified drought years as a period of a 3-year drought (2013–2015) or 2-year extreme drought (2014–2015). The solid black line represents the flooded portion of survey units during a non-drought year.

Discussion

Our study revealed changes in the abundance and areas of concentration of shorebirds over a multiyear drought cycle from 2011 to 2016 in central California. Overall, our hypotheses were largely supported in the Central Valley, where patterns of abundance were mostly similar to patterns of habitat availability, suggesting a density-dependent response by shorebirds to reduced habitat availability over the course of this multiyear drought [39]. Our results agreed with predictions of changes in habitat suitability for non-breeding shorebirds in response to the effects of drought in interior and coastal regions of southeastern Australia [40]. However, we found only minimal support for hypotheses of lower abundance of each individual focal species across all land cover types, and most did not shift areas of concentration to coastal regions. Evidence of lower abundance within both interior and coastal regions for yellowlegs and dowitchers suggests high vulnerability to the effects of drought. We found evidence of drought effects for three of four focal species in their historically preferred land cover type, and all six focal species showed evidence of drought effects within at least one cover type in the Central Valley. A notable exception was the unexpectedly high abundance of dunlin in rice fields during drought years, which was likely related to shifts in areas of concentration to remaining flooded habitats in the Sacramento Valley and Yolo-Delta sub-regions.

We found distinct patterns of relatively high shorebird abundance and habitat availability in 2012 and 2016, which were classified as non-drought years for the purposes of our study. Similar to patterns of waterbirds tracking habitat availability on broad scales across Australia [44], total shorebird abundance changed in proportion to habitat availability when considering all sub-regions in the Central Valley in 2012, and to a lesser extent in 2016. In 2012, lower habitat availability and abundance of colonial breeding herons and egrets (family Ardeidae) were

already evident in the Central Valley, most likely because four of six previous winters had less than average precipitation [45]. Despite a long-term climatic drought that was ongoing in 2012, we documented the highest annual estimate of abundance of shorebirds and habitat availability across all sub-regions and in the Sacramento Valley and Tulare sub-regions; we also documented maximum annual estimates of abundance in 2012 for the black-necked stilt, dowitchers, and five of eight non-focal species detected during our surveys. We attributed the relatively high abundance of non-breeding shorebirds in 2012 to high habitat availability resulting from a lack of curtailments to deliveries of fresh water to flood wetland and agricultural lands in the Central Valley and seasonal precipitation during the survey period. In 2016, which was the first year of average precipitation after the most extreme years of drought in 2014 and 2015, we noted an estimate of abundance of total shorebirds in the Central Valley that was similar to 2013 despite habitat availability being similar to 2011. This difference in abundance and habitat availability was most evident in the Sacramento Valley and Tulare sub-regions, suggesting a lag in response of non-breeding shorebirds repopulating some flooded habitats. Conversely, habitat availability in the Yolo-Delta and San Joaquin sub-regions in 2016 was slightly lower than in 2015, yet estimates of abundance of total shorebirds were higher than average. Overall, differences in shorebird abundance and habitat availability in the Central Valley suggest that an annual abundance estimate may be influenced by abundance during the previous year, and differences at the sub-regional scale in 2016 highlight the limitations of annual surveys for shorebirds at local scales.

Evidence of lower abundance of total shorebirds during drought than in non-drought years in the Central Valley was mostly related to declines in abundance of killdeer, black-necked stilt, and dowitchers. Our results mostly supported previous findings on habitat use patterns of

focal shorebirds in the Central Valley, including black-necked stilts preferring wetlands, and killdeer and dunlin preferring agricultural lands; however, our results suggested higher use of wetlands by yellowlegs than previously reported [21]. In our study, the killdeer and black-necked stilt were the only focal species with evidence of drought effects within the cover type in which a majority were detected. These two species were also the only focal species whose annual breeding productivity may have been directly affected by the drought, as both species breed and winter within central California, and there is evidence that their breeding populations declined from 2004 to 2013 [41]. Similarly, there is evidence that productivity of colonial breeding waterbirds in the San Francisco Bay was influenced by both extreme wet and dry climatic conditions [42]. In the Central Valley from 1978-1979 to 2013-2014, there is evidence of a declining trend for wintering populations of the killdeer and a substantial increase in wintering populations of the black-necked stilt [43]. However, whether the effects of drought had long term impacts on population trends of either species is not clear. Nonetheless, the stilt may be an ideal indicator of drought effects within managed wetlands of the Central Valley given the species' preference for wetlands and vulnerability to the effects of drought revealed in our study. Their large body size also makes them relatively easy to count.

Despite evidence of drought effects on abundance of the killdeer and black-necked stilt in the Central Valley, neither species showed evidence of shifting areas of concentration to the San Francisco Bay or Point Reyes regions. However, in the Carrizo Plain, an interior basin approximately 80 km west of Kern NWR in the Tulare sub-region, past research suggested a higher density of killdeer from 2013 to 2015 in proximity of a relatively small, natural akali wetland complex [5], suggesting that some killdeer may rely on natural fresh water wetlands as drought refugia. Our estimates of abundance of killdeer may have also been influenced by

movements within regions or sub-regions as local habitat conditions changed during wet and dry years in areas beyond our survey units, given their use of miscellaneous habitats that we did not survey extensively. Yellowlegs showed marginally lower density within rice fields during the extreme drought years of 2014 and 2015, after a maximum annual density estimate in rice in 2013. Additional evidence of significantly lower density during extreme drought years in the San Francisco Bay region is concerning given past evidence of minimal variation in annual density of greater yellowlegs during winter [27]. Moreover, in the Point Reyes region, there was a declining trend of abundance of greater yellowlegs in Bolinas Lagoon [30].

Evidence of shifts in areas of concentration of shorebirds were most substantial for populations of dunlin, and to a lesser extent least sandpiper. As the drought intensified during 2013 and 2014, the areas of concentrations of total shorebirds shifted northward in the Central Valley, where the climate is wetter and more freshwater was available for flooding. This resulted in higher abundance of dunlin despite a continued decline in flooding of survey units. By the final and most severe year of the drought in 2015, we estimated the lowest annual abundance of total shorebirds and all individual focal species in the Central Valley; habitat availability was also lowest in 2016, most likely because mandated freshwater deliveries to wetlands and agricultural lands reached minimum levels during the multiyear drought across California [47]. Models of abundance in coastal regions found evidence of higher abundance during drought years when compared to non-drought years for only the dunlin and least sandpiper. We found no evidence of increases for other focal species, and rather evidence of lower abundance of both yellowlegs and dowitchers in San Francisco Bay. Interspecies differences in patterns of abundance and areas of concentration over a drought cycle support past assertions that the loss of habitat within a region cannot be entirely mitigated with increased habitat availability in another

region in central California [23]; our results, however, highlight the likelihood that some shorebirds using interior habitats may benefit from additional coastal habitats during drought.

Our map of annual relative density of total shorebirds and our models on land cover types indicated that certain areas served as drought refugia during earlier stages of the drought in the Central Valley. Examples of drought refugia included wetlands of the Grasslands Ecological Area in the San Joaquin sub-region in 2013, and rice fields near the Sacramento River including those in the Yolo bypass in 2014. Large aggregations of shorebirds in 2014 near the Yolo bypass were notable because our estimate of habitat availability in the Yolo-Delta sub-region was the lowest in 2014, and the complex of wetlands and flooded agricultural lands that comprises the Yolo bypass is a geographic bottleneck that serves as a movement corridor for wintering waterbirds between the Sacramento Valley and Yolo-Delta sub-regions [18,46]. Our results suggest that in 2014 the costs of increased competition for resources from larger aggregations were still lower than those associated with moving to the coast, and that in 2015 a threshold was passed whereby those relative costs switched, and some shorebirds (dunlin, least sandpiper) moved to the coast.

Dunlin showed evidence suggesting broad-scale shifts of areas of concentration during drought from interior to coastal habitats in both San Francisco Bay and Point Reyes. Contrary to our prediction, our models of abundance of dunlin indicated no differences during drought years within rice fields, where >70% of their detections occurred. The large aggregations of dunlin encountered in 2014 resulted in an estimate of annual density that was around five times higher than in the other drought years of 2013 and 2015. These large aggregations likely indicated that some dunlin gathered in remaining flooded rice fields during drought years rather than redistributing to coastal habitats. The apparent broad-scale shifts in the areas of concentration of

dunlin in our study suggests high levels of mixing of regional wintering populations, which supports findings of genetic homogeneity among dunlin overwintering along the Pacific coast of North America [48]. While the ability of some birds to shift regional areas of concentrations has benefits, utilizing unfamiliar or less preferred habitat and food resources, such as shifting from interior flooded rice fields to coastal wetlands, likely has costs that may include more niche overlap with other shorebirds [49], and potentially increased risk of predation [50].

We also found evidence suggesting that some least sandpipers moved from interior to coastal sites during drought years when compared to non-drought years. Our models suggested that drought effects caused lower abundance for least sandpipers in wetlands and other suitable habitats in the Central Valley, and higher abundance in Point Reyes. Our results in the Point Reyes region supported previous studies that found cumulative seasonal rainfall during winter was correlated with the density of least sandpiper (and most other focal shorebirds) within Bolinas Lagoon [30] and Tomales Bay [29]. It is also possible that the large numbers of least sandpipers in San Francisco Bay may have masked movement from the Central Valley, where our estimates of abundance were an order of magnitude lower than in San Francisco Bay. Combined, the responses of the dunlin and least sandpiper suggest resilience to drought on broad scales, but the long-term effects, if any, on population size remain unclear. Our results also suggest that any individual conservation action may have similar effects on both the dunlin and least sandpiper, whereas the effects on other focal species may vary.

From 2013 to 2015, the effects of drought on dowitcher populations were widespread across central California, an important wintering area for non-breeding long-billed dowitchers [51]. Dowitchers were the only focal species with evidence of lower abundance in drought than in non-drought years across the Central Valley and within both wetlands and rice fields. We also

found evidence that did not support our hypothesis of dowitcher abundance being higher during drought years in San Francisco Bay; the suggestive evidence of lower abundance of dowitchers during extreme drought when compared to in non-drought years was especially surprising when considering the high fidelity to San Francisco Bay exhibited by most radio-tagged dowitchers in a past study [23]. These results suggest that dowitcher populations may be highly vulnerable to the effects of drought, unless many birds moved beyond our study area, or other factors resulted in lower abundance during drought years. More information on long-term population trends is needed for both dowitcher species across central California and for other portions of their wintering range because both dowitcher species, as well as the dunlin, are considered highly imperiled [52].

We also acknowledge limitations and logistical constraints of surveying for shorebirds over an extended time periods at a broad scale. For example, we utilized annual surveys to ensure survey long-term coverage over a broad scale, but most shorebird surveys occur at least twice per season or year to capture variability in the use of space and habitats by shorebirds. Surveying over a broad scale also resulted in variability in the total annual area surveyed because access was limited by road conditions or other logistical constraints at some survey units each year. Uneven sampling among sub-regions in the Central Valley limited our conclusions related to differences in flooding and abundance among sub-regions. Lastly, obtaining accurate counts of large aggregations of shorebirds is challenging even for highly experienced surveyors. Thus, we suspect that counts of aggregations of shorebirds in San Francisco Bay may have been less accurate than those in the Central Valley and Point Reyes because aggregations are considerably larger on average in San Francisco Bay; aggregations are especially large around high tide when

most tidal mudflats are covered by sea water. Despite these limitations we remain confident that the conclusions of our study are robust.

Conclusions

A multiyear drought substantially affected the distribution and abundance of non-breeding shorebirds and their habitats in central California, although the effects of drought on individual species varied by land cover type and geographic location. Geographic patterns of shorebird abundance showed evidence of shifting areas of concentration to northern sub-regions in the Central Valley or to the coast where habitat availability remained relatively stable during drought years. Since the 1990s, conservation efforts in the Central Valley have focused on flooding wetlands and agricultural land for non-breeding shorebirds and less effort has focused on managing for permanent wetland habitats that will be tolerant to future droughts, which may be of higher magnitude than the record breaking drought during this study. Long-term planning efforts are needed to create, restore, or expand habitats as drought refugia in the Central Valley. On the coast tidal mudflats are drought tolerant, and recent broad-scale restoration and management of wetlands within San Francisco Bay has shown potential to increase carrying capacity for shorebirds within this region of high importance to shorebirds [53].

The possibility of longer and more severe droughts indicates that a considerable investment is needed to sufficiently prepare for drought response efforts in central California. Shortfalls in available food resources for shorebirds are likely to occur in the Central Valley when habitat availability is not sufficient [31]. Effective conservation strategies should consider the amount of habitat available in the Central Valley during December 2014 [12] to be a minimum threshold below which there may be widespread declines in shorebird abundance

across all sub-regions in the Central Valley. Additionally, because many non-breeding shorebirds rely on both interior and coastal habitats in central California, coordinated management of flooded habitats for shorebirds is needed in response to future droughts to offset the potentially adverse effects of drought.

Our study agrees with others that have noted that both long-term and dynamic conservation investments are needed to increase the amount of shorebird habitats that will be reliably flooded during years of extreme drought [54]. The effects of drought on shorebirds were substantial despite legally mandated but reduced deliveries of fresh water to refuge wetlands, government funded incentive programs to flood wetlands and agricultural lands, and targeted shallow flooding for shorebirds in the Central Valley [55]. Additional flooding of managed wetlands and agricultural lands will likely be necessary to compensate for reduced habitat availability during drought years [56], and post-harvest flooded rice fields hold a high potential for increased strategic provision of freshwater habitats in central California. Locations should be prioritized that are within proximity of a complex of habitats with high likelihood of flooding or if they have the potential to provide a stepping stone between wetland complexes [18]. Additional resources for waterbirds during drought are most needed in the Tulare sub-region, which had the greatest reductions in freshwater deliveries for flooding wetlands of any sub-region in the Central Valley during drought [47]. Shorebirds would also benefit from wider implementation of recommendations of shallow-water flooding practices for agricultural lands [57] and for managed wetlands [58]. Lastly, our results provide further evidence of a highly connected network of interior and coastal wetlands across central California, and demonstrate how conservation within our study area promotes a healthy functioning network of wetlands across the Pacific Coast of the Americas.

Acknowledgments

This study was made possible by contributions from a network of government agencies, conservation foundations and organizations, and volunteers. Government agency contributions were led by Matthew Fries, Geoffrey Grisdale, Cheryl Strong, and Mike Wolder of the U.S. Fish and Wildlife Service, David Press and Matthew Lau of the National Park Service, Tanya Graham and Stacy Moskal of the U.S. Geological Survey, Laura Cockrell, Orlando Rocha, Lara Sparks, and Dave VanBaren of California Department of Fish and Wildlife, and Chris Conard of the Sacramento Regional Sanitation District Water Resources. We also thank Ben Pearl of San Francisco Bay Bird Observatory, Nils Warnock and Emiko Condeso of Audubon Canyon Ranch, Ric Ortega of Grassland Water District, Sara Sweet of The Nature Conservancy, Samantha Arthur, Meghan Hertel and Andrea Jones of Audubon California, and Kristin Sesser, Mark Dettling, and Gary Page of Point Blue Conservation Science for their contributions. Most importantly we thank the many other individuals not listed here, primarily volunteers whose tireless contributions made this study possible. This is a product of the Pacific Flyway Shorebird Survey and Migratory Shorebird Project and is Point Blue contribution number XXXX.

References

1. Duncan C, Chauvene ALM, McRae LM, Pettorelli N. Predicting the Future Impact of Droughts on Ungulate Populations in Arid and Semi-Arid Environments. PLoS One. 2012; 7(12): e51490. doi: 10.1371/journal.pone.0051490

2. Trenberth KE, Dai A, van der Schrier G, Jones PD, Barichivich J, Briffa KR, Sheffield J. Global warming and changes in drought. Nature Climate Change. 2013; 4:17-22. doi: 10.1038/s41558-018-0140-y
3. Maron M, McAlpine CA, Watson JEM, Maxwell S, Barnard P. Climate- induced resource bottlenecks exacerbate species vulnerability: a review. Diversity and Distributions. 2015; doi: 10.1111/ddi.12339
4. Maxwell SL, Butt N, Maron M, McAlpine CA, Chapman S, Ullmann A, et al. Conservations implications of ecological responses to extreme weather and climate events. Diversity and Distributions. 2019; 25:613-625. doi: 10.1111/ddi.12878
5. Prugh LR, Deguines N, Grinath JB, Suding KN, Bean WT, Stafford R, Brashares JS. Ecological winners and losers of extreme drought in California. Nature Climate Change. 2018; 8:819-824. doi:10.1038/s41558-018-0255-1
6. Albright TP, Pidgeon AM, Rittenhouse CD, Clayton MK, Flather CH, Culbert PD, Wardow BD, Radeloff VC. Effects of drought on avian community structure. Global Change Biology. 2010; 16(8):2158-2170. doi: 10.1111/j.1365-2486.2009.02120.x
7. Bennett JM, Nimmo DG, Clarke RH, Thomson JR, Cheers G, Horrocks GF, et al. Resistance and resilience: can the abrupt end of extreme drought reverse avifaunal collapse? Diversity and Distributions. 2014; 20(11):1321-32. doi: 10.1111/ddi.12230
8. Bond NR, Lake PS, Arthington AH. The impacts of drought on freshwater ecosystems: an Australian perspective. Hydrobologia. 2008; 600(1):3-16. doi: 10.1007/s10750-008-9326-z
9. Adams WM, Small RDS, Vickery JA. The impacts of land use change of migrant birds in the Sahel. Biodiversity. 2014; 15(2-3):101-108. doi: 10.1080/14888386.2014.931822

10. Griffin D, Anchukaitis KJ. How unusual is the 2012-2014 California drought? Geophysical Research Letters. 2014; 41(24):9017-9023. doi: 10.1002/2014GL062433

11. Swain DL, Langenbrunner B, Neelin JD, Hall A. Increasing precipitation volatility in twenty-first-century California. Nature Climate Change. 2018; 8:427-433. doi: 10.1038/s41558-018-0140-y

12. Reiter MA, Elliot NK, Jongsomjit D, Golet GH, Reynolds MD. Impact of extreme drought and incentive programs on flooded agriculture and wetlands in California's Central Valley. PeerJ 6:e5147. 2018; doi: 10.7717/peerj.5147

13. Fleskes JP. Wetlands of the Central Valley of California and Klamath Basin. U.S. Geological Survey. 2012; Available from: https://pubs.er.usgs.gov/publication/70157116

14. Petrie MJ, Fleskes JP, Wolder MA, Isola CR, Yarris GS, Skalos DA. Potential Effects of Drought on Carrying Capacity Potential Effects of Drought on Carrying Capacity for Wintering Waterfowl in the Central Valley of California. Journal of Fish and Wildlife Management. 2016; 7(2):408-422. doi: 10.3996/082015-JFWM-082

15. Taft OW, Colwell MA, Isola CR, Safran RJ. Waterbird responses to experimental drawdown: implications for the multispecies management of wetland mosaics. Journal of Applied Ecology. 2002; 39(6):987-1001. doi: 10.1046/j.1365-2664.2002.00763.x

16. Elphick CS, Oring LW. Winter management of Californian rice fields for waterbirds. Journal of Applied Ecology. 1998; 35(1):95-108. doi:10.1046/j.1365-2664.1998.00274.x

17. Cady SM, O'Connell TJ, Loss SR, Jaffe NE, Davis CA. Species- specific and temporal scale- dependent responses of birds to drought. Global Change Biology. 2019; 25(8):269-2702. doi: 10.1111/gcb.14668

18. Barbaree BA, Reiter ME, Hickey CM, Elliot NK, Schaffer-Smith D, Reynolds MD, Page GW. Dynamic surface water distributions influence wetland connectivity within a highly modified interior landscape. Landscape Ecology. 2018. 33(5):829-844. doi: 10.1007/s10980-018-0638-8
19. Barbaree BA, Reiter ME, Hickey CM, Page GW. Day and Night Habitat Associations of Wintering Dunlin (Calidris alpina) within an Agriculture-Wetland Mosaic. Waterbirds. 2015. 38(1):40-46. doi: 10.1675/063.038.0106
20. Matchett EL, Fleskes JP. Projected Impacts of Climate, Urbanization, Water Management, and Wetland Restoration on Waterbird Habitat in California's Central Valley. PLoS ONE. 2017; 12(1): e0169780. doi: 10.1371/journal.pone.0169780
21. Shuford WD, Page GW, Kjelmyr JE. Patterns and Dynamics of Shorebird Use of California's Central Valley. The Condor: Ornithological Applications. 1998; 100(2):227-244. doi: 10.2307/1370264
22. Robinson JA, Oring LW. Long-distance movements by American Avocets and Black-necked Stilts. Journal of Field Ornithology. 1996; 67(2):307-320. doi: jstor.org/stable/4514112
23. Takekawa JY, Warnock N, Martinelli GM, Miles AK, Tsao DC. Waterbird use of bayland wetlands in the San Francisco Bay estuary: movements of long-billed dowitchers during the winter. Waterbirds. 2002; 25:93-105.
24. Warnock N, Page GW, Stenzel LE. Non-Migratory Movements of Dunlins on Their California Wintering Grounds. The Wilson Bulletin. 1995; 107(1):131-139. doi: jstor.org/stable/4163518

25. Page GW, Stenzel LE, Kjelmyr JE. Overview of Shorebird Abundance and Distribution in Wetlands of the Pacific Coast of the Contiguous United States. The Condor. 1999; 101(3):461-471. doi: 10.2307/1370176

26. Dettinger M, Cayan DR. Drought and the California Delta—A Matter of Extremes. San Francisco Estuary and Watershed Science. 2014; 12(2). doi: 10.15447/sfews.2014v12iss2art4

27. Stenzel LE, Hickey CM, Kjelmyr JE, Page GW. Abundance and distribution of shorebirds in the San Francisco Bay area. Western Birds. 2002. 33(2):69-98.

28. Shuford WD, Page GW, Evens JG, Stenzel LE. Seasonal abundance of waterbirds at Point Reyes: a coastal California perspective. Quarterly Journal of Western Field Ornithologists. 1989. 20(4):137-266.

29. Kelly J. Distribution and abundance of wintering shorebirds in Tomales Bay, California: implications for conservation. Western Birds. 2001. 32(3):145-166.

30. Stenzel LE, Page GW. Trends in abundance of wintering waterbirds relative to rainfall patterns at a central California estuary, 1972–2015. In: Shuford WD, Gill Jr. RE, Handel CM, editors. Trends and traditions: Avifaunal change in western North America. Studies of Western Birds 3. Western Field Ornithologists, Camarillo, CA, USA; doi 10.21199/SWB3.13. 2018. pp. 236-257.

31. Dybala KE, Reiter ME, Hickey CM, Shuford WD, Strum KM, Yarris GS. A bioenergetics approach to setting conservation objectives for non-breeding shorebirds in California's Central Valley. San Francisco Estuary and Watershed Science. 2017; 15(1). doi: 10.15447/sfews.2017v15iss1art2

32. Stralberg D, Cameron DR, Reynolds MD, Hickey CM, Klausmeyer K, Busby SM, et al. Identifying habitat conservation priorities and gaps for migratory shorebirds and waterfowl in California. Biodiversity and Conservation. 2011; 20(1):19-40. doi: 10.1007/s10531-010-9943-5

33. Stanley TR, Skagen SK. Estimating the Breeding Population of Long-Billed Curlew in the United States. Journal of Wildlife Management. 2007; 71(8):2556-2564. doi: jstor.org/stable/4496376

34. Wood J, Page G, Reiter M, Liu L, Robinson-Nilsen C. Abundance and distribution of wintering shorebirds in San Francisco Bay, 1990-2008: Population change and informing future monitoring. Report to the Resource Legacy Fund. PRBO Conservation Science, Petaluma, California. 2014; http://www.prbo.org/refs/files/12082_JulianWood2010.pdf

35. Pitelka FA. Geographic variation and the species problem in the shore-bird genus Limnodromus. University of California Press. 1950.

36. Murtaugh PA. In defense of P values. Ecology. 2014; 95(3):611–617. doi: 10.1890/13-0590.1

37. Zuur A, Ieno EN, Walker N, Saveliev AA, Smith GM. Mixed Effects Models and Extensions in Ecology with R. 2009; https://www.springer.com/us/book/9780387874579

38. Brooks ME, Kristensen K, van Benthem KJ, Magnusson A, Berg CW, Nielsen A, et al. glmmTMB balances speed and flexibility among packages for zero-inflated generalized linear mixed modeling. The R Journal. 2017; 9(2):378-400. Available from: https://journal.r-project.org/archive/2017/RJ-2017-066/index.html

39. Goss-Custard JD, Clarke RT, Le V. Dit Durell SEA, Caldow RWG, Ens BJ. Population Consequences of Winter Habitat Loss in a Migratory Shorebird. II. Model Predictions. Journal of Applied Ecology. 1995; 32(2):337-351. doi: 10.2307/2405100

40. Wen L, Saintilan N, Reid JR, Colloff MJ. Changes in distribution of waterbirds following prolonged drought reflect habitat availability in coastal and inland regions. Ecology and Evolution. 2016; 6(18):6672-6689. doi: 10.1002/ece3.2091

41. Strum KM, Dybala KE. Iglecia MN, Shuford WD. Population and Habitat Objectives for Breeding Shorebirds in California's Central Valley. San Francisco Estuary and Watershed Science. 2017; 15(1). doi: 10.15447/sfews.2017v15iss1art3

42. Kelly JP, Condeso TE. Rainfall Effects on Heron and Egret Nest Abundance in the San Francisco Bay Area. Wetlands. 2014; 34:893. doi:10.1007/s13157-014-0551-0

43. Pandolfino ER, Handel CM. Population trends of birds wintering the Central Valley of California. 2018. In Trends and Traditions: Avifaunal change in Western North America (Shuford WD, Gill Jr. RE, Handel, CM, eds.). Studies of Western Birds 3; Western Field Ornithologists, Camarillo, CA, USA. pp 215 - 235. doi: 10.21199/SWB3.12

44. Kingsford RT, Roshier DA, Porter JL. Australian waterbirds—time and space travellers in dynamic desert landscapes. Marine and Freshwater Research. 2010; 61:875-884. doi 10.1071/MF09088.

45. Shuford WD, Kelly, JP, Condeso TE, Cooper DS, Molina KC, Jongsomjit D. Distribution and abundance of colonial herons and egrets in California, 2009-2012. Western Filed Ornithologists. 2020; In Press.

46. Ackerman JT, Takekawa JY, Orthmeyer DL, Fleskes JP, Yee JL, Kruse KL. Spatial Use by Wintering Greater White- Fronted Geese Relative to a Decade of Habitat Change in

California's Central Valley. Journal of Wildlife Management 2006; 70(4):965-976. doi: 10.2193/0022-541X(2006)70[965:SUBWGW]2.0.CO;2\

47. Lund J, Medellin-Azuara J, Durand J, Stone K. Lessons from California’s 2012 - 2016 drought. Journal of Water Resources Planning and Management. 2018; 144(10):04018067. doi: 10.1061/(ASCE)WR.1943-5452.0000984

48. Popovic I, Toews DPL, Keever CC, St. Clair CT, Barbaree BA, Fernández G, Rourke J. Genotyping-by-sequencing reveals genomic homogeneity among overwintering Pacific Dunlin (Calidris alpina pacifica) aggregations along the Pacific coast of North America. 2019; 121:1–12. doi: 10.1093/condor/duz036

49. Catry T, Lourenço PM, Lopes RJ, Carneiro C, Alves JA, Costa J, et al. Structure and functioning of intertidal food webs along an avian flyway: a comparative approach using stable isotopes. Functional Ecology. 2015; 30(3):468-478. doi: 0.1111/1365-2435.12506

50. Lank DB, Butler RW, Ireland J, Ydenberg RC. Effects of predation danger on migration strategies of sandpipers. Oikos. 2003; 103:303-319. doi: 10.1034/j.1600-0706.2003.12314.x

51. Barbaree BA, Reiter ME, Hickey CM, Page GW. Molt migration and migratory connectivity of the long-billed dowitcher. Journal of Wildlife Management. 2016; 80(2):256-265. doi: 10.1002/jwmg.1006

52. Galbraith H, DesRochers DW, Brown S, Reed JM. Predicting Vulnerabilities of North American Shorebirds to Climate Change. PLoS One. 2014; 9(9): e108899.

53. Brand LA, Takekawa JY, Shinn J, Graham T, Buffington K, Gustafson KB, et al. Effects of wetland management on carrying capacity of diving ducks and shorebirds in a coastal estuary. Waterbirds. 2014; 37(1):52-67. doi: 10.1675/063.037.0108

54. Reynolds MD, Sullivan BL, Hallstein E, Matsumoto S, Kelling S, Merrifield M, et al. 2017. Dynamic conservation for migratory species. Science Advances 2017; doi: e1700707
55. Golet GH, Low C, Avery S, Andrews K, McColl CJ, Laney R, Reynolds MD. Using ricelands to provide temporary shorebird habitat during migration. Ecological Applications. 2018; 28(2):409-426. doi: 10.1002/eap.1658
56. Steen V, Skagen SK, Noon BR. Preparing for an uncertain future: migrating shorebird response to past climatic fluctuations in the Prairie Potholes. Ecoshpere. 2018; 9(2): e02095. doi: 10.1002/ecs2.2095
57. Strum KM, Reiter ME, Hartman CA, Iglecia MN, Kelsey TR, Hickey CM. Winter management of California's rice fields to maximize waterbird habitat and minimize water use. Agriculture, Ecosystems & Environment. 2013; 179(1):116-124. doi: 10.1016/j.agee.2013.08.003
58. Schaffer- Smith D, Swenson JJ, Reiter ME, Isola JE. Quantifying shorebird habitat in managed wetlands by modeling shallow water depth dynamics. Ecological Applications. 2018; 28(6):1534-1545. doi: 10.1002/eap.1732

Supporting information

S1 Figure. The annual mean flooded portion of shorebird survey units by land cover type. Annual means with error bars representing 95% confidence intervals from data collected during shorebird surveys from 2011 to 2016 in the Central Valley, California, USA. Survey units are categorized by the dominant land cover type within unit boundaries. The dashed line represents the annual mean estimate across all cover types. We defined “other suitable habitats” as those

with potential for use by shorebirds when flooded, including grasslands/pasture, corn fields, other irrigated row or field crops, freshwater lakes/ponds, and wastewater treatment ponds. We defined "miscellaneous" land cover types as having low potential for use by shorebirds, and included developed, forested, riverine, orchard, and vineyard.

S2 Figure. The annual mean flooded portion of shorebird survey units by sub-region in the Central Valley.

Annual means with error bars representing 95% confidence intervals from data collected during shorebird surveys from 2011 to 2016 in the Central Valley, California, USA. Survey units are categorized by geographic location within sub-regions. The dashed line represents the annual mean estimate across all cover types.

S1 Table. Annual density of total shorebirds (per km^2) and survey effort across central California, USA.

Estimated density of total shorebirds (per km^2) among four sub-regions of the Central Valley and two coastal regions from 2011 to 2016 across central California, USA. We did not estimate the mean density of total shorebirds within coastal regions. Summary of our annual survey effort is located under each density estimate and displayed as the number of units and total area surveyed (km^2).

[1] No survey at Los Banos Wildlife Area in 2014.

[2] No survey at Kern or Pixley National Wildlife Refuge in 2011 and 2014.

[3] No survey of Suisun Marsh in 2011.

[4] No surveys of Drakes Estero or nearby waters in 2011 and 2012.

S2 Table. Annual density of total shorebirds (per km^2) and survey effort in the Central Valley.

Estimated density of total shorebirds (per km^2) among four land cover types from 2011 to 2016 in the Central Valley, California, USA. Summary of our annual survey effort is located under each density estimate and displayed as the number of units and total area surveyed (km^2). We defined "other suitable habitats" as those with potential for use by shorebirds when flooded, including grasslands/pasture, corn fields, other irrigated row or field crops, freshwater lakes/ponds, and wastewater treatment ponds. We defined "miscellaneous" land cover types as having low potential for use by shorebirds, and included developed, forested, riverine, orchard, and vineyard.

S3 Table. Annual density (per km^2) of focal shorebirds and mean total count in the Central Valley.

Annual density estimates among four dominant land cover types from 2011 to 2016 in the Central Valley, California, USA. Mean annual counts are the average annual survey totals for each species without accounting for the total area surveyed.

S4 Table. Annual density (per km2) of non-focal shorebirds and mean count in the Central Valley.

Annual density estimates from 2011 to 2016 in the Central Valley, California, USA. Mean annual counts are the average annual survey totals for each species without accounting for the total area surveyed.

S5 Table. Annual density (per km^2) of focal shorebirds and mean total count in central California, USA.

Annual density estimates from 2011 to 2016 among three regions in central California, USA. Mean annual counts are the average annual survey totals for each species without accounting for the total area surveyed.

S6 Table. Results of models of annual abundance of focal shorebirds in central California, USA.

Results of generalized linear mixed models of abundance of six focal shorebirds among three regions of central California, USA. Data collected during annual shorebird surveys from 2011 to 2016. Models tested differences in abundance between non-drought years and drought years (2013 to 2015 for drought models; 2014 to 2015 for extreme drought models). Parameter estimates (β) and 95 % confidence intervals (CI) are back-transformed to the scale of the response variable. *P*-values are from a null-hypothesis likelihood-ratio test on the t-statistic for each parameter. Statistically significant p-values are in italics. Deviance scores estimate the goodness of fit for each model; a lower score represents better goodness of fit.

S7 Table. Results of models of annual abundance of total shorebirds in central California, USA.

Results of generalized linear mixed models of abundance of total shorebirds four land cover types in the Central Valley and among three regions of central California, USA. Data collected during annual shorebird surveys from 2011 to 2016. Models tested differences in abundance between non-drought years and drought years (2013 to 2015 for drought models; 2014 to 2015 for extreme drought models). Parameter estimates (β) and 95 % confidence intervals (CI) are back-transformed to the scale of the response variable. *P*-values are from a null-hypothesis likelihood-ratio test on the t-statistic for each parameter. Statistically significant p-values are in

italics. Deviance scores estimate the goodness of fit for each model; a lower score represents better goodness of fit.

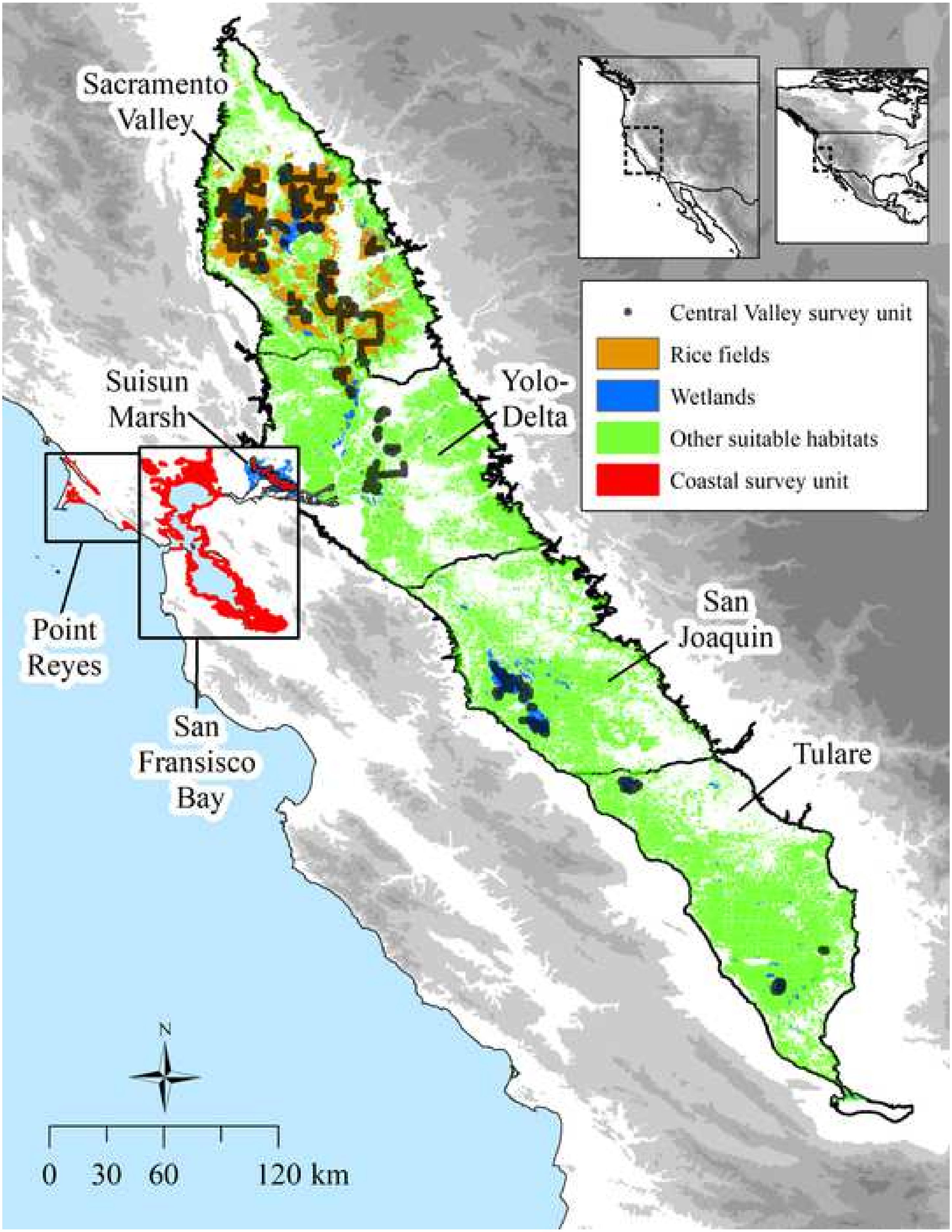
Sacramento Valley
Suisun Marsh
Yolo-Delta
Point Reyes
San Fransisco Bay
San Joaquin
Tulare
Central Valley survey unit
Rice fields
Wetlands
Other suitable habitats
Coastal survey unit
N
0 30 60 120 km

Figure 2

Click here to access/download;Figure;Fig 2.tif

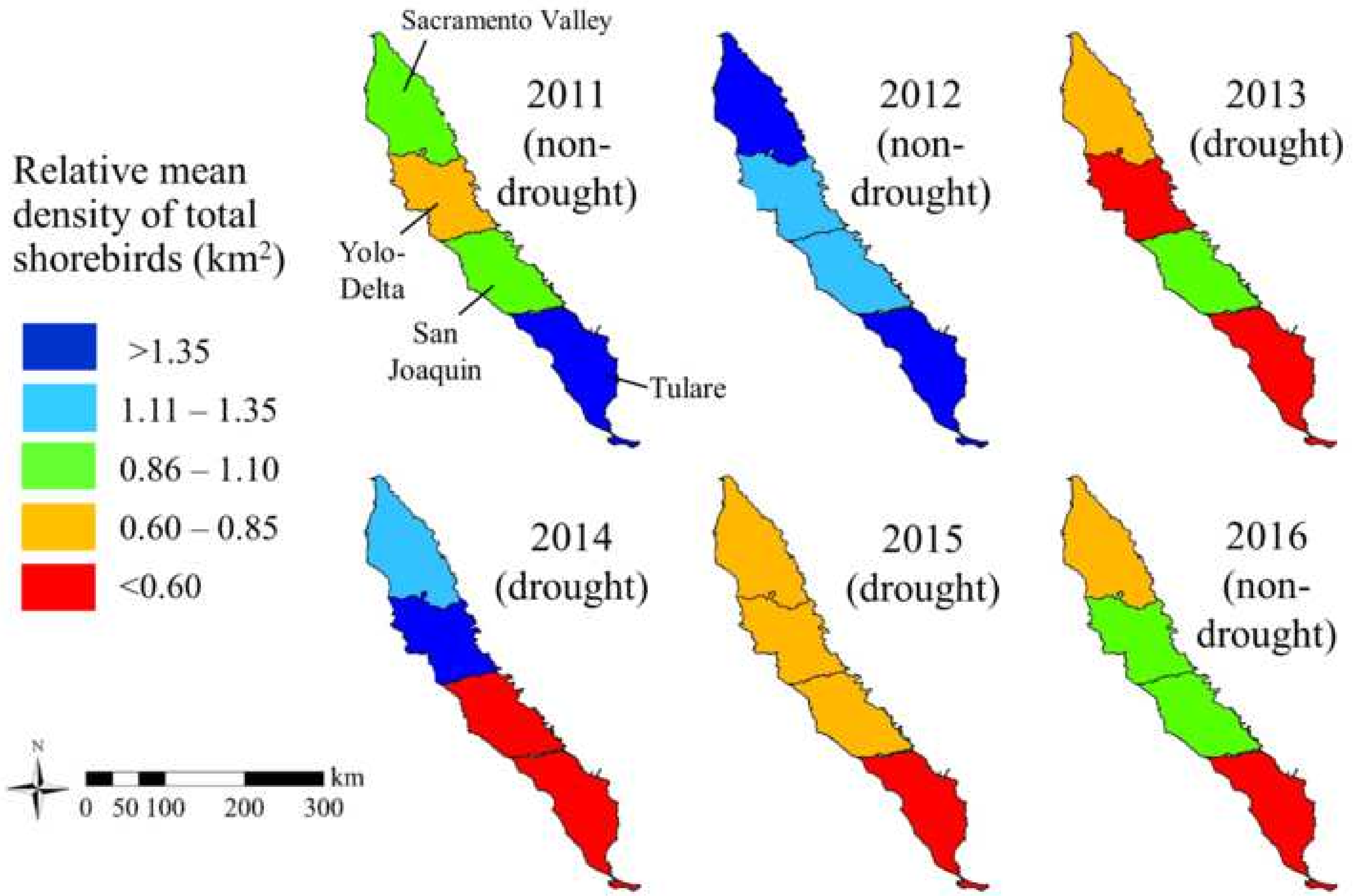

Figure 3

Click here to access/download;Figure;Fig 3.tif

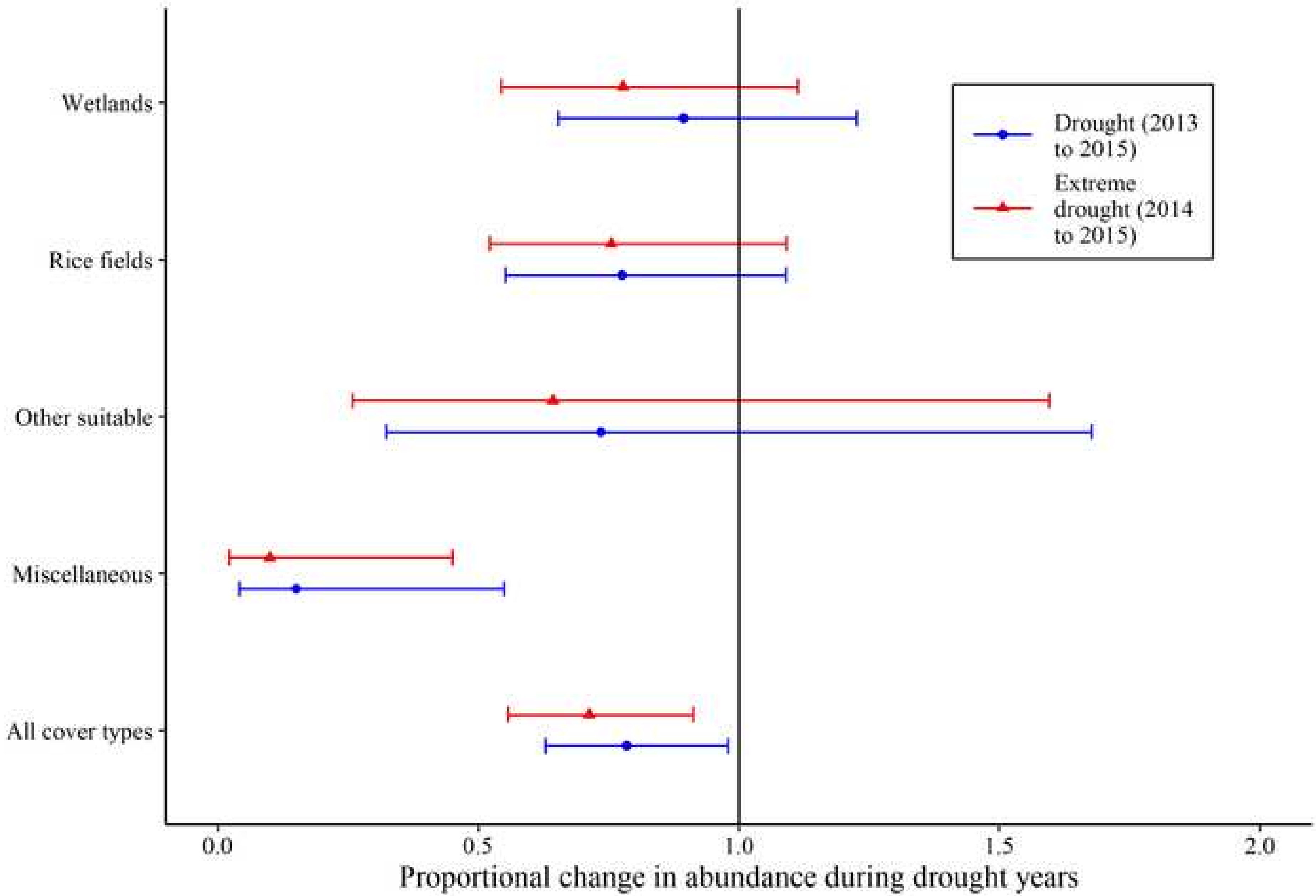

Figure 4

Click here to access/download;Figure;Fig 4.tif

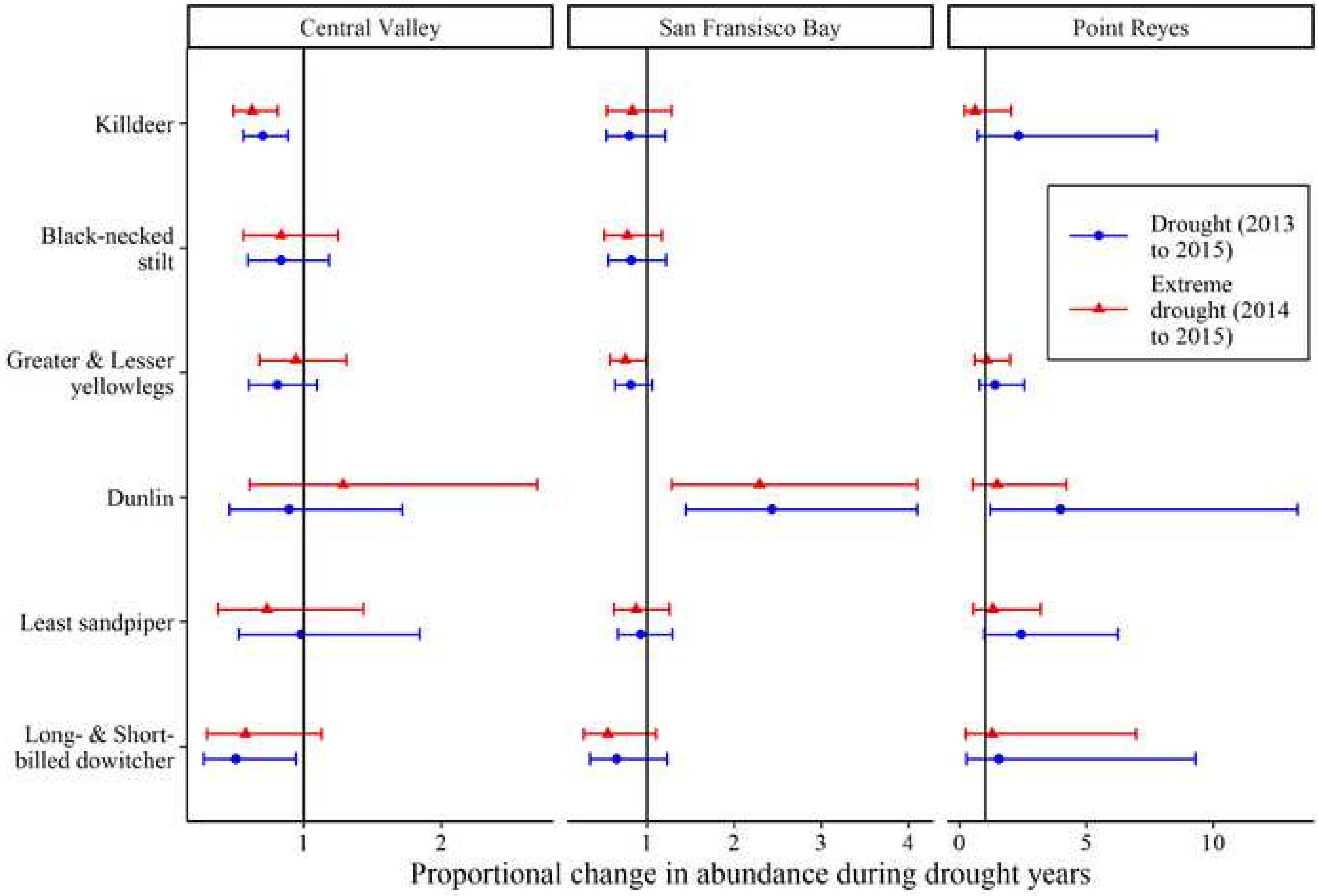

Figure 5

Click here to access/download;Figure;Fig 5.tif

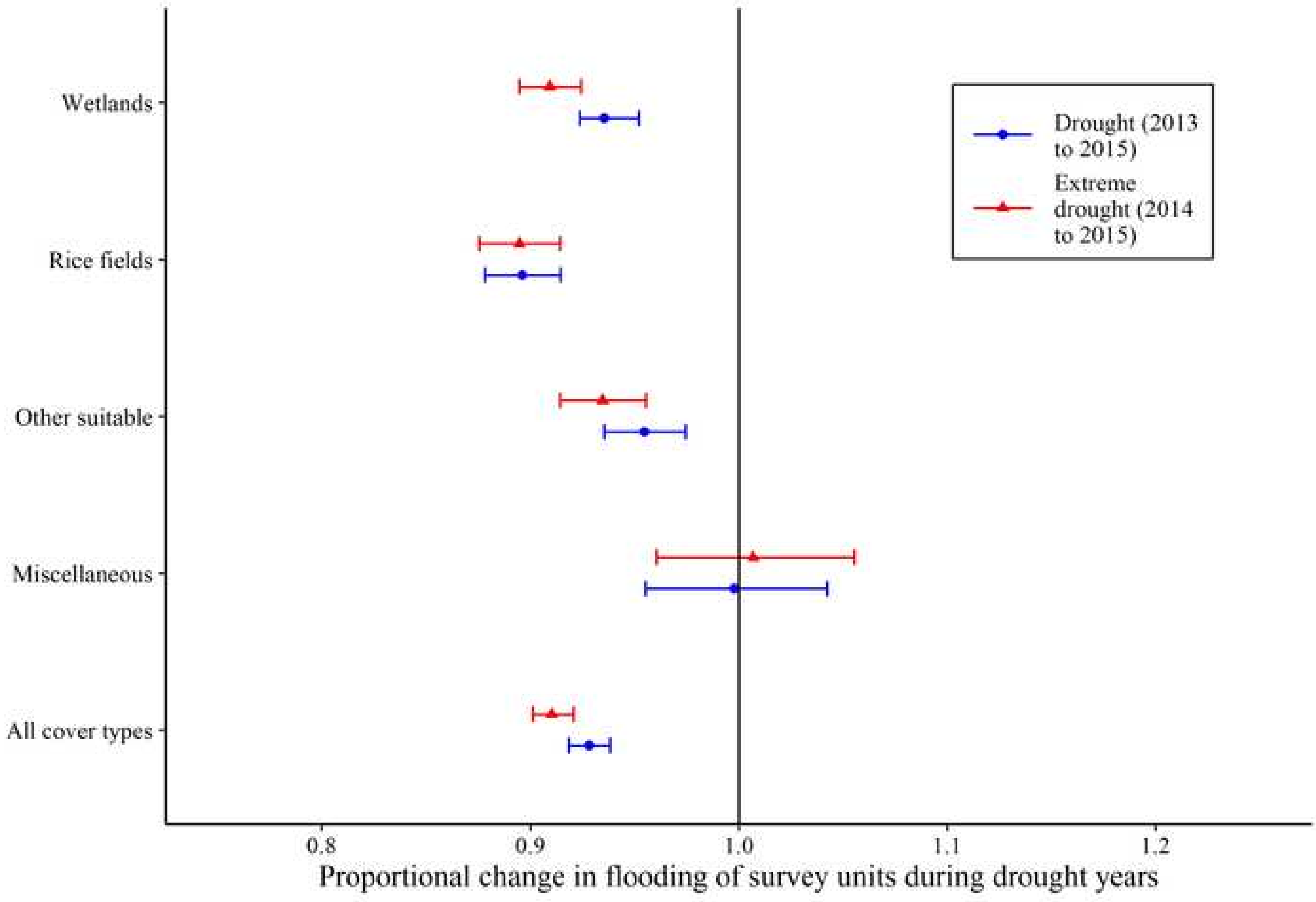

S1 Figure

Click here to access/download;Figure;S1 Fig.tif

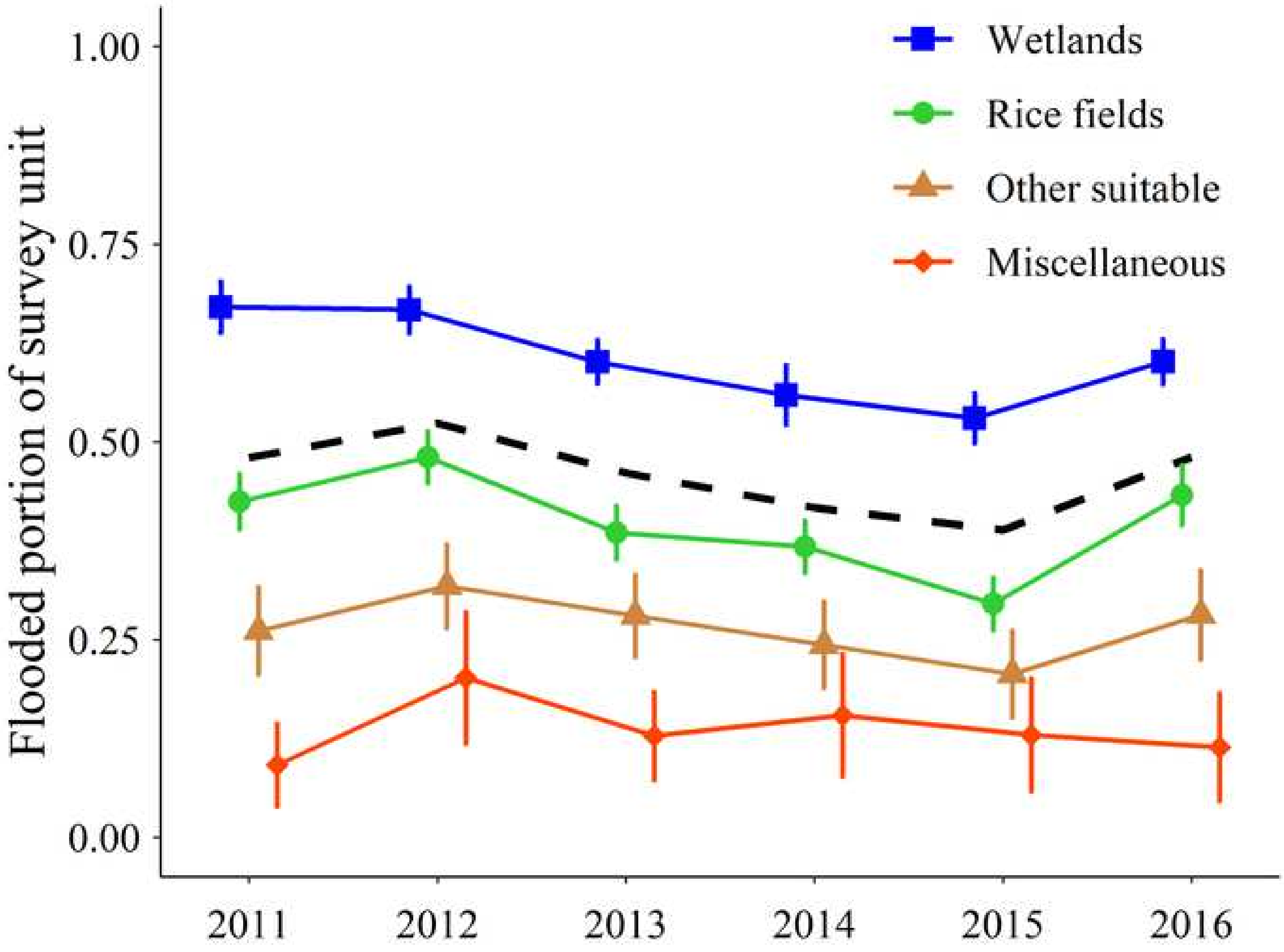

Click here to access/download;Figure;S2 Fig.tif

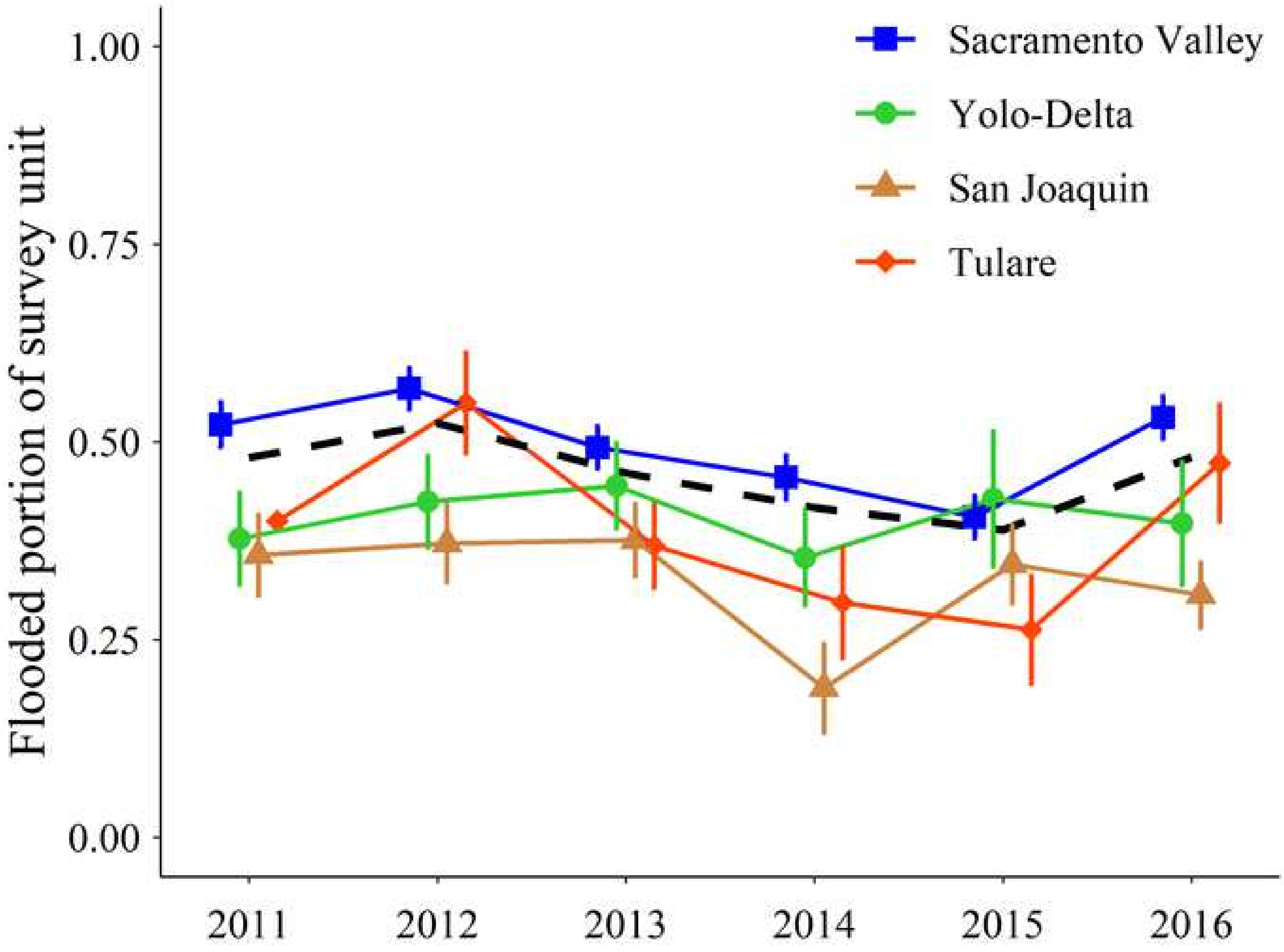

Supporting information tables

Click here to access/download
Supporting Information
S1-7 Tables.docx
