# Supplementary material for: Effects of drought on the abundance and distribution of non-breeding shorebirds in central California, USA
Source: PLoS One. 2020 Oct 21;15(10):e0240931. doi: 10.1371/journal.pone.0240931 (PMC7577470; doi:10.1371/journal.pone.0240931)
Supplement: Attachment — Submitted filename: S1-7 Tables_reviewer comments.docx [file pone.0240931.s013.docx]

# **Supporting information**

**S1 Table. Annual density of total shorebirds (per km^2^) and survey effort across central California, USA.**

Estimated density of total shorebirds (per km^2^) among four sub-regions of the Central Valley and two coastal regions from 2011 to 2016 across central California, USA. We did not estimate the mean density of total shorebirds within coastal regions. Summary of our annual survey effort is located under each density estimate and displayed as the number of units and total area surveyed (km^2^).

^1^ No survey at Los Banos Wildlife Area in 2014.

^2^ No survey at Kern or Pixley National Wildlife Refuge in 2011 and 2014.

^3^ No survey of Suisun Marsh in 2011.

^4^ No surveys of Drakes Estero or nearby waters in 2011 and 2012.

**S2 Table. Annual density of total shorebirds (per km^2^) and survey effort in the Central Valley.**

Estimated density of total shorebirds (per km^2^) among four land cover types from 2011 to 2016 in the Central Valley, California, USA. Summary of our annual survey effort is located under each density estimate and displayed as the number of units and total area surveyed (km^2^). We defined “other suitable habitats” as those with potential for use by shorebirds when flooded, including grasslands/pasture, corn fields, other irrigated row or field crops, freshwater lakes/ponds, and wastewater treatment ponds. We defined “miscellaneous” land cover types as having low potential for use by shorebirds, and included developed, forested, riverine, orchard, and vineyard.

**S3 Table. Annual density (per km^2^) of focal shorebirds and mean total count in the Central Valley.** Annual density estimates among four dominant land cover types from 2011 to 2016 in the Central Valley, California, USA. Mean annual counts are the average annual survey totals for each species without accounting for the total area surveyed.

**S4 Table. Annual density (per km2) of non-focal shorebirds and mean count in the Central Valley.**

Annual density estimates from 2011 to 2016 in the Central Valley, California, USA. Mean annual counts are the average annual survey totals for each species without accounting for the total area surveyed.

**S5 Table. Annual density (per km^2^) of focal shorebirds and mean total count in central California, USA.**

Annual density estimates from 2011 to 2016 among three regions in central California, USA. Mean annual counts are the average annual survey totals for each species without accounting for the total area surveyed.

**S6 Table. Results of models of annual abundance of focal shorebirds in central California, USA.**

 Results of generalized linear mixed models of abundance of six focal shorebirds among three regions of central California, USA. Data collected during annual shorebird surveys from 2011 to 2016. Models tested differences in abundance between non-drought years and drought years (2013 to 2015 for drought models; 2014 to 2015 for extreme drought models). Parameter estimates (β) and 95 % confidence intervals (CI) are back-transformed to the scale of the response variable. *P*-values are from a null-hypothesis likelihood-ratio test on the t-statistic for each parameter. Statistically significant p-values are in italics. Deviance scores estimate the goodness of fit for each model; a lower score represents better goodness of fit.

**S7 Table. Results of models of annual abundance of total shorebirds in central California, USA.**

Results of generalized linear mixed models of abundance of total shorebirds across four land cover types in the Central Valley and among three regions of central California, USA. Data collected during annual shorebird surveys from 2011 to 2016. Models tested differences in abundance between non-drought years and drought years (2013 to 2015 for drought models; 2014 to 2015 for extreme-drought models). Parameter estimates (β) and 95 % confidence intervals (CI) are back-transformed to the scale of the response variable. *P*-values are from a null-hypothesis likelihood-ratio test on the t-statistic for each parameter. Statistically significant p-values are in italics. Deviance scores estimate the goodness of fit for each model; a lower score represents better goodness of fit.
